# Systems biology of electrogenic *Pseudomonas putida* - multi-omics insights and metabolic engineering for enhanced 2-ketogluconate production

**DOI:** 10.1186/s12934-024-02509-8

**Published:** 2024-09-11

**Authors:** Anna Weimer, Laura Pause, Fabian Ries, Michael Kohlstedt, Lorenz Adrian, Jens Krömer, Bin Lai, Christoph Wittmann

**Affiliations:** 1 Institute of Systems Biotechnology, Saarland University, Saarbrücken, Germany; 2 Systems Biotechnology Group, Helmholtz Centre for Environmental Research - UFZ, Leipzig, Germany; 3 Department of Molecular Environmental Biotechnology, Helmholtz Centre for Environmental Research – UFZ, Leipzig, Germany; 4 BMBF Junior Research Group Biophotovoltaics, Helmholtz Centre for Environmental Research - UFZ, Leipzig, Germany

**Keywords:** *Pseudomonas putida* KT2440, Bio-electrochemical system, Systems biology, Electron transfer, Anode, 2-ketogluconate, Ribosome, Bio-production, Anoxic metabolism, Redox mediator

## Abstract

**Background:**

*Pseudomonas putida* KT2440 has emerged as a promising host for industrial bioproduction. However, its strictly aerobic nature limits the scope of applications. Remarkably, this microbe exhibits high bioconversion efficiency when cultured in an anoxic bio-electrochemical system (BES), where the anode serves as the terminal electron acceptor instead of oxygen. This environment facilitates the synthesis of commercially attractive chemicals, including 2-ketogluconate (2KG). To better understand this interesting electrogenic phenotype, we studied the BES-cultured strain on a systems level through multi-omics analysis. Inspired by our findings, we constructed novel mutants aimed at improving 2KG production.

**Results:**

When incubated on glucose, *P. putida* KT2440 did not grow but produced significant amounts of 2KG, along with minor amounts of gluconate, acetate, pyruvate, succinate, and lactate. ^13^C tracer studies demonstrated that these products are partially derived from biomass carbon, involving proteins and lipids. Over time, the cells exhibited global changes on both the transcriptomic and proteomic levels, including the shutdown of translation and cell motility, likely to conserve energy. These adaptations enabled the cells to maintain significant metabolic activity for several weeks. Acetate formation was shown to contribute to energy supply. Mutants deficient in acetate production demonstrated superior 2KG production in terms of titer, yield, and productivity. The *∆aldBI ∆aldBII* double deletion mutant performed best, accumulating 2KG at twice the rate of the wild type and with an increased yield (0.96 mol/mol).

**Conclusions:**

By integrating transcriptomic, proteomic, and metabolomic analyses, this work provides the first systems biology insight into the electrogenic phenotype of *P. putida* KT2440. Adaptation to anoxic-electrogenic conditions involved coordinated changes in energy metabolism, enabling cells to sustain metabolic activity for extended periods. The metabolically engineered mutants are promising for enhanced 2KG production under these conditions. The attenuation of acetate synthesis represents the first systems biology-informed metabolic engineering strategy for enhanced 2KG production in *P. putida*. This non-growth anoxic-electrogenic mode expands our understanding of the interplay between growth, glucose phosphorylation, and glucose oxidation into gluconate and 2KG in *P. putida*.

**Supplementary Information:**

The online version contains supplementary material available at 10.1186/s12934-024-02509-8.

## Background

*Pseudomonas putida* is commonly found in diverse environments due to its highly adaptable metabolism [1, 2]. Among its strains, *P. putida* KT2440 is widely used in laboratories and industrial biotechnology as a safe and reliable cell factory [3–7]. Research efforts have expanded *P. putida*’s natural capabilities by integrating custom synthetic properties through genomic modifications [3, 4], which include enabling an anoxic lifestyle [8–11]. By nature, *P. putida* KT2440 is a strictly aerobic bacterium, lacking fermentation and anaerobic respiration pathways [2]. However, this strain can catalyze biochemical reactions under anoxic conditions using an anode as the terminal electron acceptor [12, 13]. This anodic electron transfer can involve mediator molecules either produced by the cells [14] or added externally [12], with the latter facilitating much higher electron transfer rates.

When cultivated in an anaerobic bio-electrochemical system (BES), *P. putida* produces different products depending on the substrate used [13–16]. With glucose, the microbe primarily produces 2-ketogluconate (2KG) via periplasmic oxidation. Commercially, 2KG is of relevance in the food, cosmetics, pharmaceutical, and environmental industries [17]. Similarly, various other aldoses are oxidized to their corresponding (keto-)aldonates [15]. Due to the importance of periplasmic oxidation in this metabolic mode, it has been extensively studied [16, 18]. Interestingly, *P. putida* also generates several metabolic by-products in addition to periplasmic (keto-)aldonates [12]. These by-products, originating from the cytosolic Embden-Meyerhof-Parnas (EMP) pathway and the tricarboxylic acid (TCA) cycle, indicating a more complex metabolism than simple periplasmic substrate oxidation. For example, electrogenic *P. putida* accumulates small amounts of acetate extracellularly [18].

*P. putida* KT2440 lacks the acetate kinase (*ackA*) gene found in facultative anaerobic relatives like *P. aeruginosa* but contains genes such as *aldBI/II* and *scpC*, which may be involved in acetate formation [8, 10, 19, 20]. Previous studies under anoxic conditions have shown mixed results regarding the benefits of heterologous *ackA* expression for survival [8, 10, 20]. Notably, acetate and pyruvate are not common products but have been reported in response to (p)ppGpp accumulation [21] and in mutants lacking the global regulator Crc [22] which exhibit artificial metabolic overflow due to the saturation of catabolic enzymes and deregulation of substrate uptake [23].

In this study, we investigated the anoxic electrogenic phenotype of *P. putida* KT2440 at a systems level. We applied multi-omics profiling during the bio-electrochemical oxidation of glucose, integrating the analysis of metabolites, proteins, and transcripts to uncover previously unexplored metabolic reactions and pathways active under these conditions. Glucose was predominantly oxidized in the periplasm but also served as a substrate for synthesizing cytosolic organic and amino acids. Stable isotope studies revealed that *P. putida* KT2440 recycled cell resources to generate low molecular weight metabolites, mainly acetate. We found that acetate was partially remobilized from cellular lipids via β-oxidation to acetyl-CoA units. Analysis of deletion mutants, each lacking one of four potential biosynthetic pathways, showed that acetate synthesis involved the *aldBI/II* and *scpC* routes. Remarkably, reduced acetate formation in the *∆aldBI ∆aldBII* and *∆scpC* mutants was coupled with drastically accelerated glucose oxidation. Comprehensive changes at the transcriptome and proteome levels indicated a globally coordinated adaptation of *P. putida* to the bio-electrochemical conditions over time, suggesting the cells had adjusted their metabolic activity to a new homeostatic state.

## Results

**Fig. 1:**
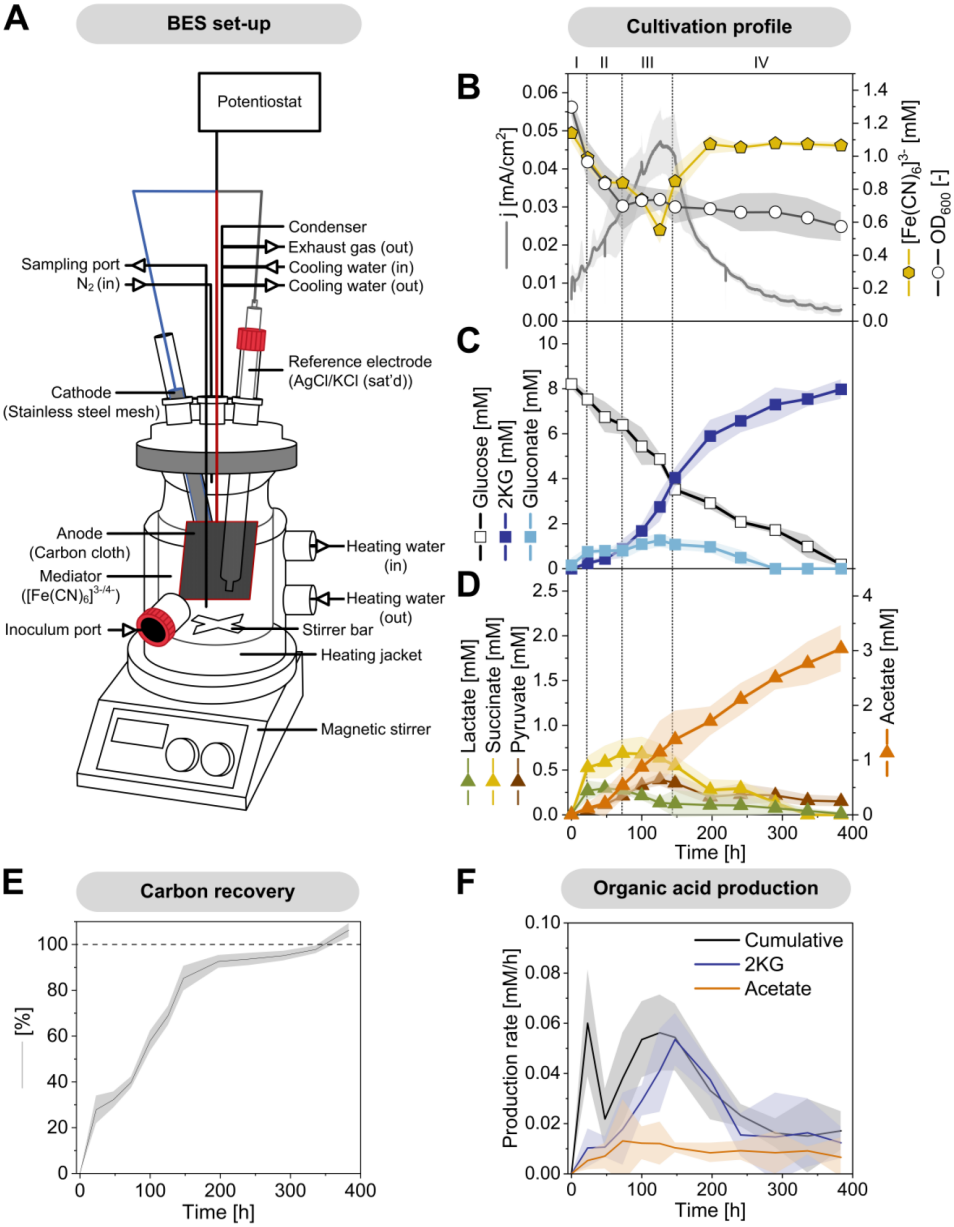
Bio-electrochemical fermentation of *P. putida* KT2440 on glucose. The bio-electrochemical reactor system (BES) comprised to compartments: The liquid volume of the anodic compartment containing the cultured cells was 320 mL. Ferricyanide, [Fe(CN)_6_]^3−^, was added to the medium as redox mediator. The reactor was operated at 500 rpm and 30 °C and sparged with N_2_ at a flow rate of 2 L h^− 1^. The potential of the working electrode was set to 0.5 V against the reference electrode (Ag/AgCl, in saturated KCl) (A). The data comprise the time profiles of current density (mA/cm^2^), cell concentration (OD_600_), and [Fe(CN)_6_]^3−^ (mM) (B), glucose (mM), gluconate (mM), 2-ketogluconate (mM) (C), and other organic acids (mM) (D). The data were corrected for evaporation effects (Additional file 1) and used to estimate the carbon balance (E), as well as the yields and specific production rates of 2-ketogluconate, acetate, and cumulative acid production over time, respectively (F), *n* = 4

**Fig. 2:**
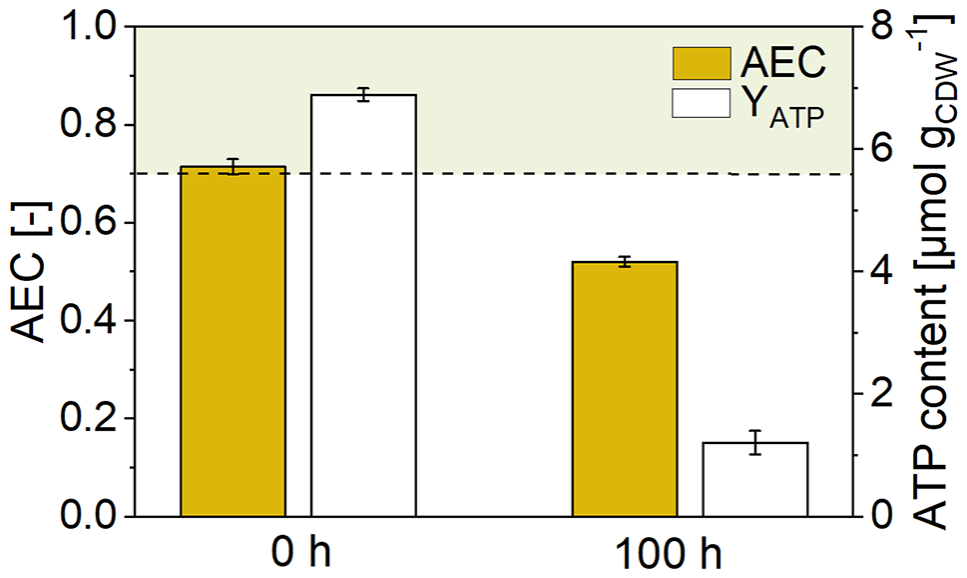
Changes in energy metabolism during bio-electrochemical fermentation of *P. putida* KT2440 on glucose. The data show the ATP content (µmol g_CDW_^−1^) and the adenylate energy charge (AEC) after 0 h and 100 h

***Pseudomonas putida *KT2440 forms different mono- and dicarboxylic acids when cultured on glucose in an oxygen-free anodic bio-electrochemical system (BES).** This study investigated the metabolic activities of *P. putida* in lab-scale BES reactors equipped with an anode to capture electrons generated by anoxic glucose oxidation [12] (Fig. 1A). The minimal medium was supplemented with the external electron mediator ferricyanide ([Fe(CN)_6_]^3-^) to enable electron transfer. The conversion of glucose in the BES took approximately 380 h (Fig. 1B), resulting in the production of gluconate, 2KG, pyruvate, lactate, acetate, and succinate to varying extents (Fig. 1C and D). Gluconate and 2KG were produced via the periplasmic glucose oxidation route [24], while pyruvate, lactate, acetate, and succinate originated from cytosolic pathways. Interestingly, the ATP content after 100 h (1.2 ± 0.02 µmol g_CDW_⁻¹) was significantly lower than that of cells analyzed at the process start (6.9 ± 0.10 µmol g_CDW_⁻¹). Despite this, the anoxic-electrogenic metabolism established an adenylate energy charge (AEC) of 0.52 ± 0.01 (Fig. 2), which, while lower than at the process start, remained above the values observed for oxygen-starved *P. putida* cells [10].

Shortly after inoculation into the BES, the cells rapidly adapted to the electrochemical environment, initiating electron current generation. The process proceeded through four distinct phases, each characterized by different metabolic activities (Fig. 1B, C, D and F). During phase I (0–24 h), all observed products were released into the medium, and cell concentration decreased. During phase II (24–75 h), succinate production ceased, lactate was re-consumed, and the formation of gluconate, 2KG, acetate, and pyruvate continued. Current generation increased while cell concentration further decreased. In phase III (75–140 h), cells displayed the highest electrochemical activity. Cell concentration stabilized, and significant amounts of gluconate, 2KG, and acetate were produced. Peak current density reached 0.047 mA/cm² (± 0.007). During phase IV (140–380 h), current generation decreased. Gluconate, succinate, and pyruvate were re-consumed, while 2KG and acetate production continued. Throughout the process, both oxidized and reduced forms of the mediator were present (Fig. 1B). Initially, the mediator was fully oxidized (OD_420_ = 1.13 ± 0.04, equivalent to 1.14 mM oxidized mediator). At peak current, approximately 50% of the mediator was reduced (0.55 ± 0.07 mM), suggesting limitations of diffusion in the bulk liquid and oxidation at the anode surface as electron generation increased.

A stoichiometric inspection of the process revealed several notable aspects. Based on the degree of reduction of glucose and the formed products, the overall electron balance (104.9 ± 2.7%) did not completely close. This imbalance suggested that apparently more electrons were captured by the anode than could be theoretically accounted for by the conversion of glucose to metabolites. The primary product, 2KG, was obtained at a concentration of 7.9 mM, corresponding to a molar yield of 88.4% (0.88 mol mol^− 1^ glucose). The temporal dynamics of current generation closely matched the specific rate of 2KG formation (Fig. 1B and F), indicating that 2KG production was the primary pathway for anodic electron transfer. The second most abundant product, acetate, reached a concentration of 3.0 mM, with a yield of 0.34 mol mol^− 1^ glucose. However, the stoichiometry of the pathways indicated that the available glucose was insufficient to synthesize both 2KG and acetate. The formation of 2KG consumed 88.4% of the glucose substrate, while the additional acetate formation would have required another 16.9% of the substrate (accounting for one CO₂ molecule formed per acetate), implying that only a portion of the acetate could derive from glucose. This observation was further supported by the calculation of carbon recovery in extracellular products. At the end of the process, the recovered carbon exceeded the amount available from glucose (106.3 ± 2.6%) (Fig. 1E). This discrepancy suggests that additional carbon sources, likely from biomass breakdown, contributed to product formation. This hypothesis aligns with the observed decrease in cell concentration (Fig. 1B) and recent findings that indicate biomass degradation in BES-cultured *P. putida* serves as a carbon source [18]. Furthermore, the chemical composition of the cells changed during the process. The carbon-to-nitrogen (C: N) ratio increased from 3.44 ± 0.01 at the process start to 3.87 ± 0.03 after 100 h, indicating significant metabolic adaptations during bio-electrochemical fermentation (Fig. 4A).

**Fig. 3:**
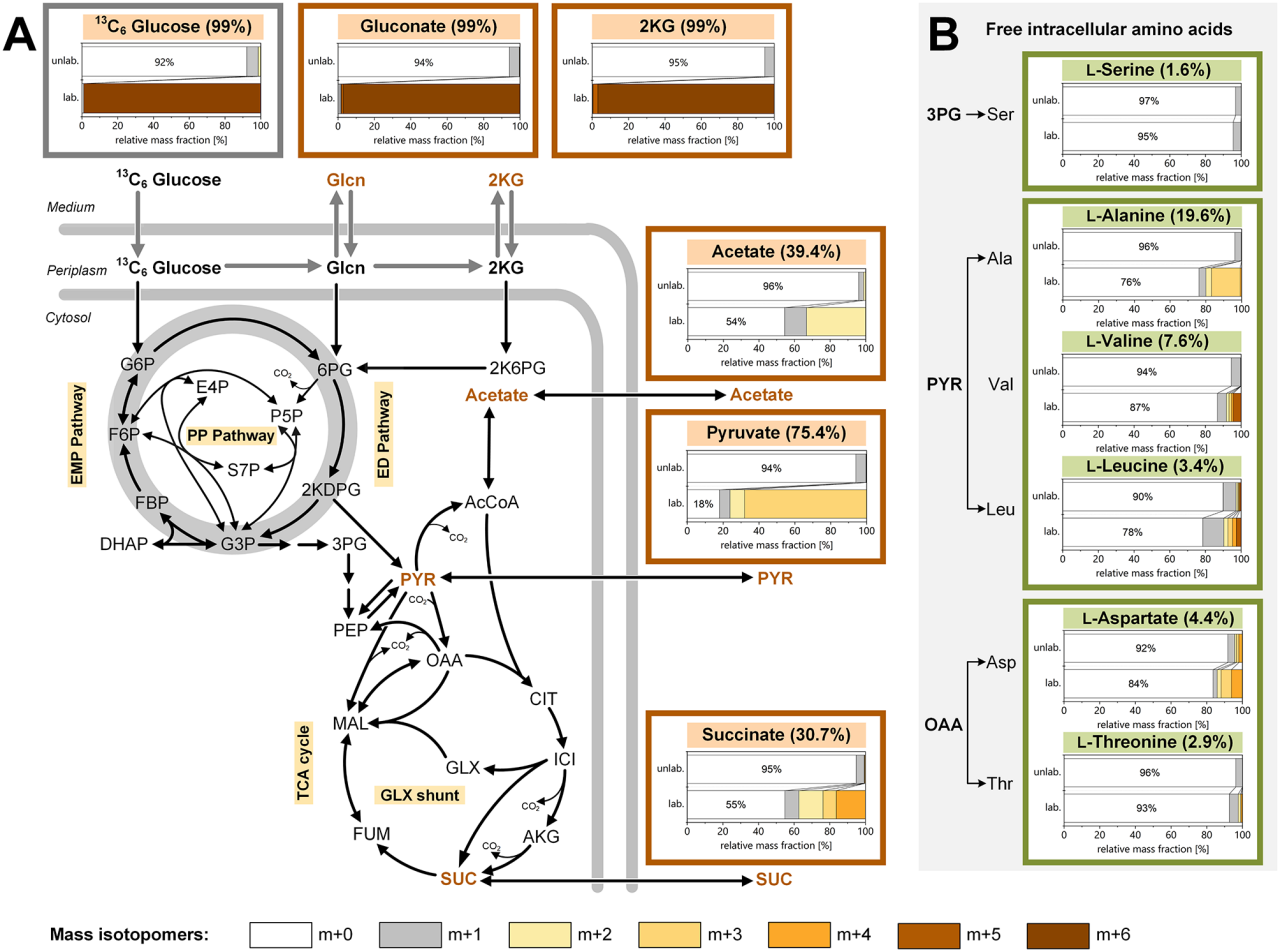
Isotopic profiling of anoxic-electrogenic *P. putida* KT2440 grown on [^13^C_6_] glucose. The data display the ^13^C enrichment of extracellular accumulated organic acids (A) and intracellular amino acids (B), sampled after 100 h from the bio-electrochemical process using [^13^C_6_] glucose as the substrate. For comparison, the BES process was conducted using naturally labelled glucose, i.e. non-^13^C-labelled glucose, as the substrate. The bar graphs represent the measured mass isotopomer distributions of the analytes (m + 0 to m + x) using [^13^C_6_] glucose (lower bars, lab.) and non-^13^C-labelled glucose (upper bars, unlab.). The numbers given above the bar charts display the summed fraction labelling from the tracer study on [^13^C_6_] glucose, calculated after correction of the measured labelling data for natural isotope abundance. As shown, gluconate and 2-ketogluconate exclusively originate from glucose, while the other organic acids partially stem from the [^13^C_6_]-labelled glucose and (non-labelled) biomass constituents. Amino acids are largely derived from biomass

**Fig. 4:**
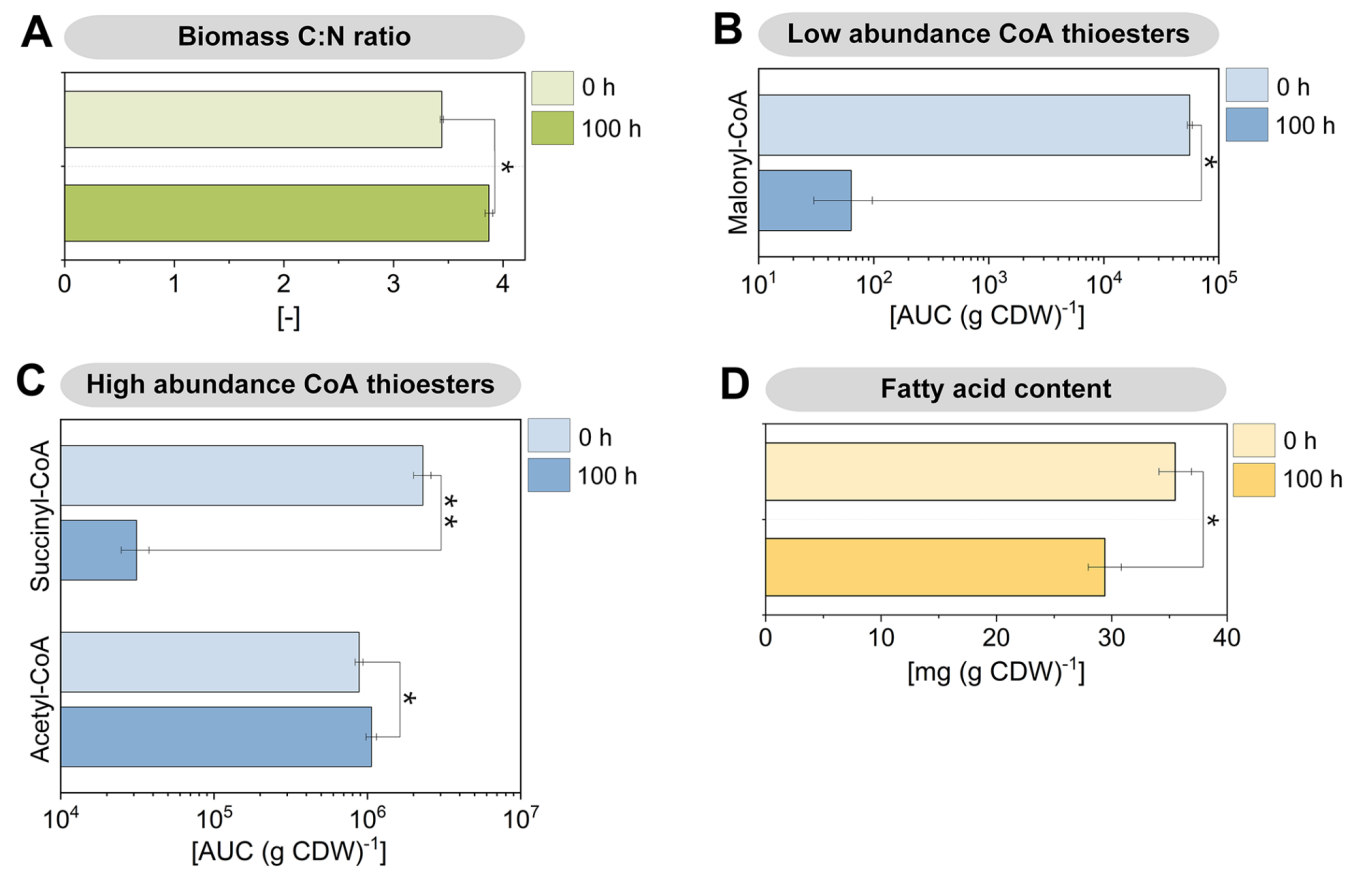
Metabolomic changes during bio-electrochemical fermentation of *P. putida *KT2440 on glucose. The data show the C:N ratio of the biomass (A), the abundance of intracellular CoA thioesters (B, C), and the fatty acid content of the biomass after 0 h and 100 h. The significance of differences between the time points is indicated as follows: * (*p* < 0.05) and ** (*p* < 0.01). *n* = 3

**Isotopic **^**13**^**C tracer studies reveal the diverse metabolic origins of extracellular organic acids and the continuation of amino acid and protein synthesis in bio-electrochemically cultured *P. putida***. In the anodic bio-electrochemical process, *P. putida* produced six different organic acids. To determine their biosynthetic origins, we used ^13^C isotope profiling [25]. For this purpose, a bio-electrochemical fermentation was conducted using 99% [^13^C_6_] glucose, inoculated with cells from a non-labelled pre-culture, and sampled after 100 h for GC-MS and LC-MS ^13^C labelling analysis of 2KG, gluconate, acetate, pyruvate, and succinate. Although lactate was not present at the sampling point, its precursor, pyruvate, provided its labelling information. The summed fraction labelling (SFL) data indicated the relative abundance of ^13^C atoms in the formed acids, showing the extent to which these acids contained carbon from glucose (^13^C) or biomass (^12^C) (Fig. 3A). Gluconate and 2KG were fully ^13^C enriched, confirming they were synthesized de novo from the provided glucose, whereas acetate, pyruvate, and succinate incorporated biomass carbon, consistent with the observed decrease in cell concentration at the beginning of the process (Fig. 1B and D).

Pyruvate exhibited a summed fraction labelling (SFL) of 75.4%, indicating that glucose metabolized via the ED and lower EMP pathways was its major carbon source, with the remaining 24.6% derived from biomass breakdown. Notably, acetate (SFL = 39.4%) and succinate (SFL = 30.7%) were much less ^13^C enriched, suggesting they primarily originated from the breakdown of biomass components (Fig. 3A). Further analysis of the ^13^C labelling pattern in free intracellular amino acids after 100 h revealed that amino acids from the pyruvate and oxaloacetate families were enriched in ^13^C (Fig. 3B), indicating ongoing de novo synthesis from glucose. Alanine showed the highest SFL at 19.6%, whereas serine had low ^13^C enrichment (SFL = 1.6%) (Fig. 3B). Additionally, ^13^C labelling was detected in protein-incorporated amino acids obtained through cell protein hydrolysis (SFL 0.5–2.0%). These values were approximately ten-fold lower than those in the free intracellular pools but significantly above the threshold of natural labelling (SFL = 0.02 ± 0.02%). Considering that free-form amino acids likely constituted only about 1% of the total cellular pool, the observed ^13^C incorporation into protein biomass clearly indicated new protein synthesis (Additional file 2, Fig. S1). However, the relatively low degree of labelling suggested that only limited protein (re)synthesis occurred, potentially restricted to a specific subset of proteins.

**Acetate production during bio-electrochemical cultivation is largely fueled from biomass-derived acetyl-CoA and is linked to significant changes in lipid metabolism**. Acetate was a prominent by-product (Fig. 1D), with much of it not originating from pyruvate, which is a common precursor in glucose-grown cells. Under anoxic conditions, many microbes convert pyruvate to acetate via acetyl-CoA [26]. However, in this study, approximately half of the acetate was derived from biomass without involving pyruvate, as evidenced by its significantly lower SFL (Fig. 3A). To further understand this process, we analyzed the abundance of intracellular CoA thioesters using LC-MS/MS [27] (Fig. 4B and C). Succinyl-CoA levels were significantly lower in bio-electrochemically cultured cells compared to aerobic cells, likely due to the disrupted TCA cycle in the absence of oxygen. Additionally, the malonyl-CoA levels in cells from the BES reactor were 1000-fold lower, indicating a near absence of fatty acid synthesis, which typically begins with malonyl-CoA [28]. Interestingly, BES-cultured *P. putida* KT2440 exhibited a 20% increase in acetyl-CoA. This acetyl-CoA likely originated from fatty acid degradation via β-oxidation, as evidenced by a significant decrease in the overall fatty acid content of the cells during the bio-electrochemical process (Fig. 4D). This suggests that intracellular lipids served as a major carbon source, and the elevated acetyl-CoA levels potentially drove acetate synthesis. Furthermore, we observed changes in the fatty acid composition, with a shift from cis- to trans-configured unsaturated fatty acids, which could impact membrane fluidity [29], although the average fatty acid length remained unchanged (Additional file 2, Table S2).

**Fig. 5:**
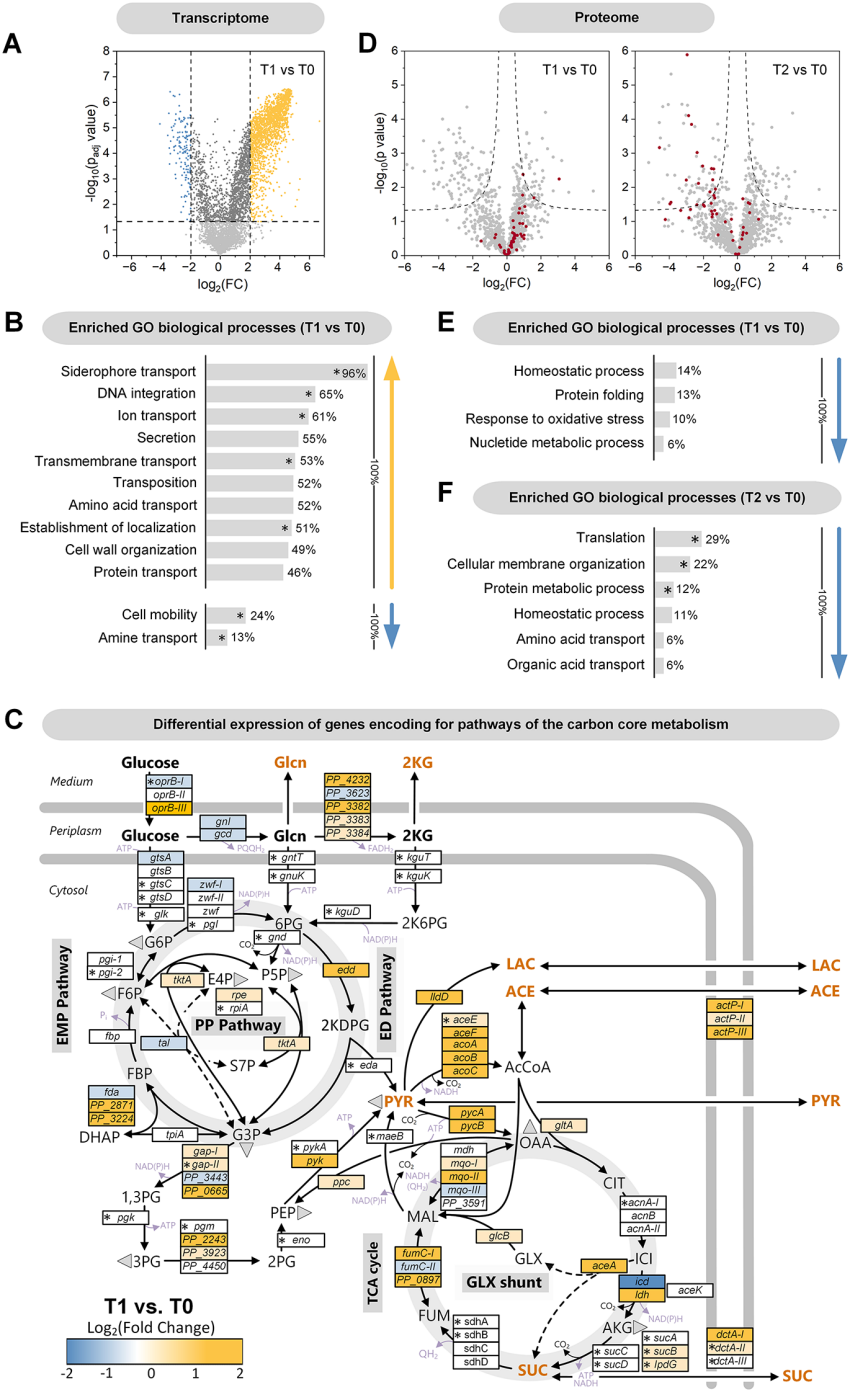
Transcriptomic and proteomic changes during bio-electrochemical fermentation of *P. putida* KT2440 on glucose. Volcano plot of global transcriptomic differences between 0 h (T0) and 24 h (T1) (A). Assignment of transcriptional changes between 0 h and 24 h to GO biological processes (Gene Ontology), *n* = 4. (B). The given percentage depicts the coverage of a category by significantly changed transcripts. Significantly enriched GO biological processes are marked * (Fisher’s exact test). Expression dynamics of genes related to the central carbon core metabolism between 0 h and 24 h (C). For completeness, non-significantly changed genes are included (marked *, Benjamini‒Hochberg FDR > 0.05), *n* = 4. Volcano plots of global proteomic changes between 0 h (T0), 24 h (T1), and 100 h (T2) (D), *n* = 3. Ribosomal proteins are labelled red. Assignment of proteomic changes between 0 h and 24 h (E), as well as 0 h and 100 h (F) to GO biological processes. The given percentage depicts the coverage of a category by significantly changed proteins. Significantly enriched GO biological processes are marked * (Fisher’s exact test)

***P. putida *KT2440 coordinates its adaptation to electrogenic conditions at the transcriptional level.** To achieve a comprehensive understanding, we investigated the extent to which transcriptional regulation facilitated adaptation to the BES environment and how this affected the protein inventory. Transcriptomic and proteomic analyses were conducted at various time points during the process. High-quality mRNA was obtained after 0, 24, and 100 h, showing high reproducibility among biological replicates (Additional file 2, Fig. S2). *P. putida* KT2440 exhibited substantial changes in gene expression throughout the process (Additional file 2, Fig. S3A). After 24 h, compared to the start of the process, 2,011 genes (36.1% of 5,564 coding sequences) were significantly upregulated (p_adj_ < 0.05, log2-fold change > 2), while 176 genes (3.2%) were significantly downregulated (p_adj_ < 0.05, log2-fold change < -2) (Fig. 5A, Additional file 2, Fig. S3). This indicates that the cells initiated a broad adaptive response to the anaerobic conditions. Upregulated genes were predominantly involved in membrane-related processes, such as transport, secretion, and cell wall organization. Conversely, downregulated genes were mainly associated with cell mobility. Gene Ontology (GO) enrichment analysis identified several biological processes that were significantly overrepresented during the initial 24-hour phase, including siderophore transport (96%), DNA integration (65%), ion transport (61%), secretion (55%), transmembrane transport (53%), transposition (52%), amino acid transport (52%), establishment of localization (51%), and cell wall organization (49%) (Fig. 5B). Conversely, categories such as cell mobility (24%) and amine transport (13%) were less enriched. Significant changes were also observed in central carbon metabolism (Fig. 5C, Additional file 2, Table S3). Genes encoding the gluconate-2-dehydrogenase complex (PP_3382, PP_3383, PP_3384) were upregulated, consistent with 2KG being the main product, whereas the unspecific subunit PP_3623 was not upregulated. Additionally, genes involved in the pyruvate node *(pycAB*,* aceF*,* acoABC*) and the glyoxylate shunt were significantly upregulated. Increased levels of acetyl-CoA have been reported to stimulate the kinase activity of isocitrate dehydrogenase kinase/phosphatase (AceK), leading to phosphorylation and partial inactivation of isocitrate dehydrogenase (Icd) [30]. In line with this, *icd* was downregulated, likely redirecting metabolic flux towards the glyoxylate shunt. Acetate symporters (*actP-I*,* actP-II*,* actP-III*) were also upregulated. Furthermore, there was a switch in expression from cytosolic NADH-forming malate dehydrogenase (*mdh*) to membrane-bound quinol-forming malate dehydrogenase (*mqo-1*,* mqo-2*), which may align with adaptations in electron transport. In electrogenic *P. putida*, cytochrome c reductase has been identified as the key enzyme for electron transfer to the mediator [Fe(CN)_6_]^3−^ [31] with quinol serving as a direct substrate for the cytochrome c reductase complex. In contrast, genes related to glucose oxidation and phosphorylation (*gtsABCD*), triose recycling (*tpiA*,* fda*,* fbp*,* pgi*), and the pentose phosphate (PP) pathway (*zwf*,* pgI*) were largely unchanged or slightly downregulated. Overall, the expression pattern remained stable during later phases, with over 80% of the upregulated genes being consistent at both the 24 and 100-hour time points. Temporal differences were more pronounced among the downregulated genes (Additional file 2, Fig. S3A).

**Fig. 6:**
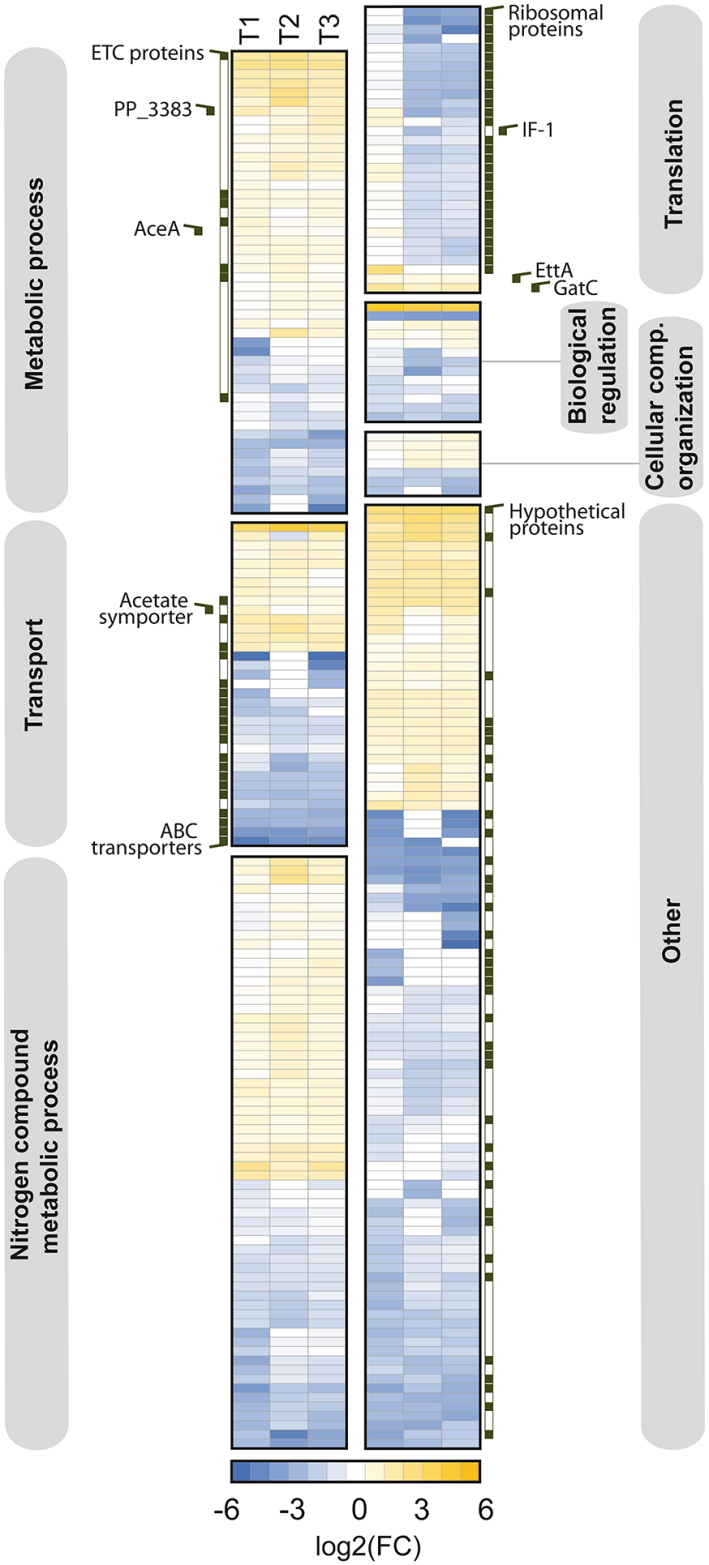
Hierarchical clustering of proteomic changes during bio-electrochemical fermentation of *P. putida* KT2440 on glucose. The heatmap depicts significant changes in the proteome between 0 h and 24 h (T1), 100 h (T2), as well as 380 h (T3), *n* = 3. Significantly changed proteins (-1 > log2-fold > 1, p value < 0.05) are grouped with one way ANOVA against the start sample, whereby the grouping is based on the corresponding Gene Ontology “biological process” annotations

**Proteome analysis reveals an early adaptation in carbon and nitrogen metabolism**,** as well as transport processes**,** and a general shutdown of translation in later stages.** The changes at the protein level were limited to comparatively few enzymes and regulators compared to the transcriptome (Fig. 5D, Additional file 2, Fig. S3B). After 24 h, the abundance of 95 proteins decreased, while the level of 40 proteins significantly increased. This indicates that the bacterium selectively adjusted its protein inventory to the anoxic-electrogenic conditions. Proteins that showed significant changes over time (24 h, 100 h, 380 h) were primarily related to carbon and nitrogen metabolism, transport processes, and translation (Fig. 6). For example, enzymes from the electron transport chain (PP_2867, NuoC, SdhA, NuoE, CyoB, Ndh) increased in abundance, likely due to their role in electron transfer to the external mediator. Additionally, the glyoxylate shunt enzyme AceA, gluconate 2-dehydrogenase (PP_3383), and the acetate symporter ActP-I were also increased, consistent with the transcriptome data. In contrast, several energy-dependent ABC transporters were less abundant. Many of the affected proteins had unknown functions (Fig. 6). The proteome analyzed after 100 and 380 h was comparable to that of the 24-hour time point, with a notable exception: proteins involved in translation were significantly affected in the later stages (Fig. 6). Until the 24-hour time point, ribosomal proteins remained stable in abundance, indicating active translation during this phase (Fig. 5D and E). After 100 h, many translation-associated proteins were less abundant, making translation a significantly enriched GO biological process with decreased associated protein levels (Fig. 5D and F). The translation initiation factor IF-1 (InfA, PP_4007) was reduced, while the level of EttA (PP_0674) increased. EttA acts as an ADP/ATP ratio sensor and translation inhibitor. Additionally, the abundance of 27 structural ribosomal proteins decreased, with reductions up to 24-fold, as observed for RpsU (PP_0389), a component of the small 30 S ribosomal subunit (Fig. 6, Additional file 3, Table S1).

**Fig. 7:**
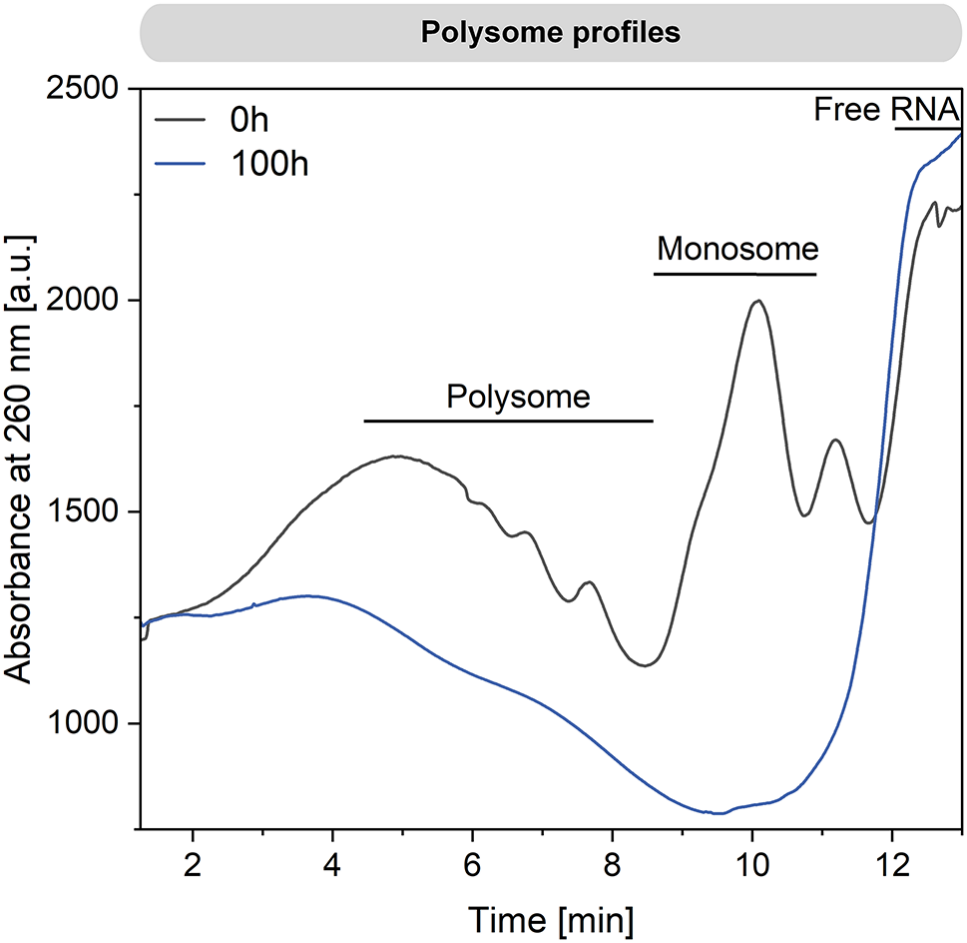
Ribosome profiling during bio-electrochemical fermentation of *P. putida* KT2440 on glucose. The data display the abundance of different ribosome variants after 0 h and 100 h

To understand how these changes impacted the functionality of the translation machinery, we conducted sucrose gradient sedimentation analysis (Fig. 7). At the start of the process, cells contained a substantial fraction of polysomes, indicating active translation. Both polysomes and monosomes, which play crucial roles in translation, were present. However, after 100 h, both forms had nearly disappeared, indicative of significantly reduced translation activity. *P. putida* KT2440 appeared to maintain a state of homeostasis over the extended fermentation period (380 h). This observation prompted a reevaluation of the transcriptome data from the later stages of the process (100 h) (Additional file 2, Fig. S4). The apparent reduction in active ribosomes suggested that mRNA levels at this point might reflect transcriptional responses that could no longer be fully translated into protein. Additionally, technical aspects might have influenced the transcriptome data after 100 h. The protocol required total RNA as input, and given that rRNA constitutes the majority of RNA in bacterial cells (80–90%) [32, 33], a reduction in the rRNA pool could have resulted in a relative increase in mRNA abundance in the samples from later process stages used for microarray analysis. This potentially led to an overestimation of gene expression. Therefore, the main conclusions on transcriptional adaptation should be primarily derived from the 24-hour sample, as done earlier.

**Fig. 8:**
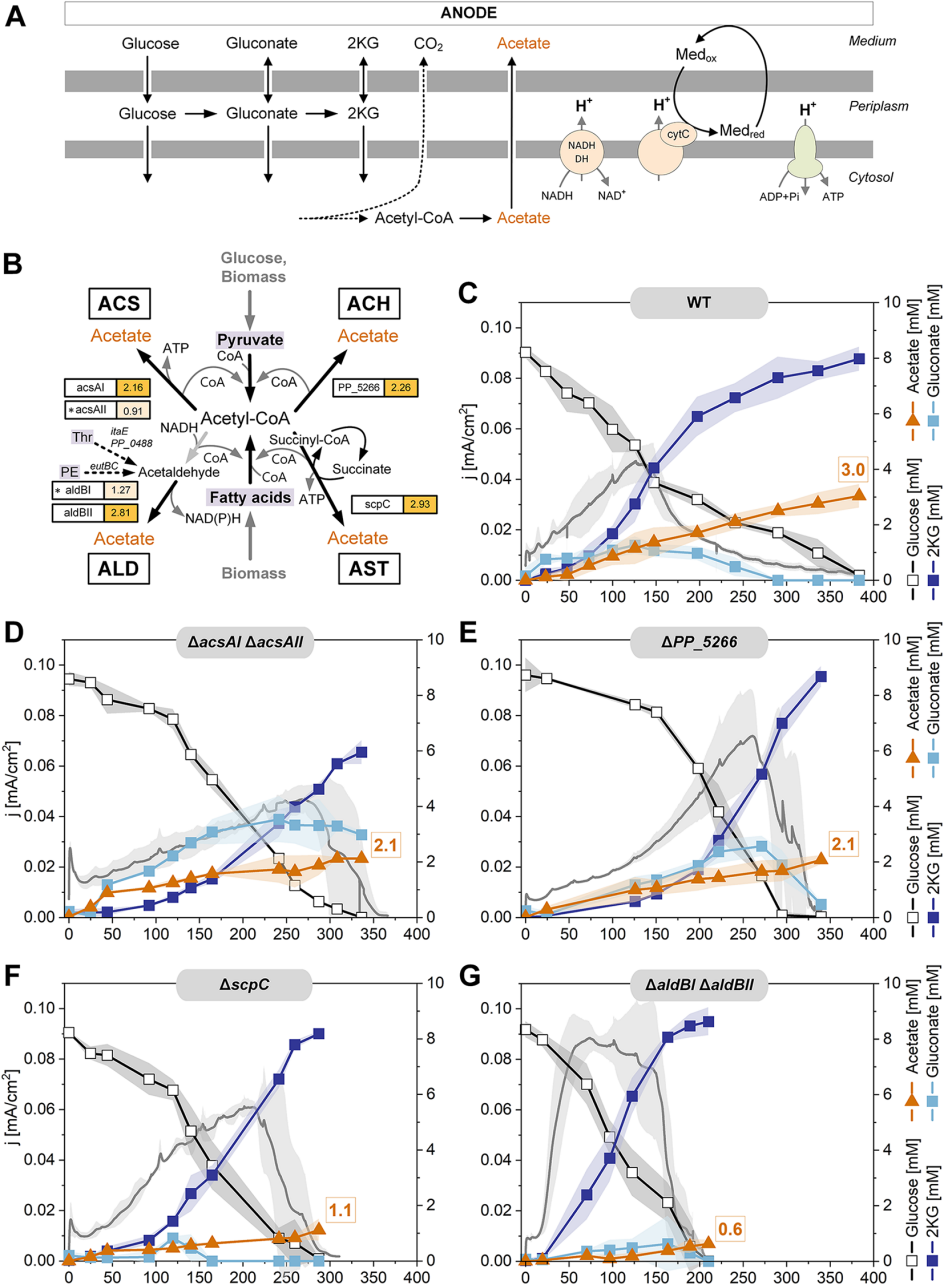
Impact of acetate production during bio-electrochemical fermentation of *P. putida* KT2440 on glucose. Schematic representation of the major energy-yielding pathways and processes under these conditions, including periplasmic glucose oxidation, cytosolic acetate formation, proton translocation by cytochrome c reductase and NADH dehydrogenase, respectively, and ATP generation by ATPase (A). Potential routes for acetate biosynthesis in *P. putida* KT2440, all originating from acetyl-CoA and connected to catabolic breakdown of glucose and biomass constituents (B). Fermentation profiles of KT2440 and deletion mutants, each lacking one of the four acetate biosynthetic routes (C-G). The data represent the current density (mA/cm^2^) and the concentrations of glucose, gluconate, 2-ketogluconate, and acetate, *n* = 4. Additional data on glucose and by-products are given in the Additional file 2

**Elucidation of the biosynthetic routes to acetate under anoxic-electrogenic conditions.** Acetate accumulated significantly under anoxic-electrogenic conditions (Fig. 1D). This biosynthesis pathway is poorly understood, as acetate is typically not synthesized aerobically. Given the substantial acetate formation under these conditions, we aimed to unravel the underlying biochemistry. *P. putida* KT2440 encodes four potential pathways for acetate biosynthesis from acetyl-CoA: (i) the acetyl-CoA synthase (ACS) pathway operating in reverse to generate ATP (ii), the ATP-independent acetyl-CoA hydrolase (ACH) pathway, (iii) the NAD(P)H-generating aldehyde dehydrogenase (ALD) pathway, and (iv) the acetate CoA-transferase (AST) pathway, which couples ATP formation through the regeneration of succinyl-CoA into succinate via succinyl-CoA synthetase (Fig. 8). To investigate these pathways, we constructed four mutants, each lacking one of the four pathways (Fig. 8B). The genes of interest were removed by in-frame deletion (Table 1). For pathways involving two enzymes, both corresponding genes were deleted sequentially. The mutant *P. putida* KT2440 *ΔacsAI ΔacsAII* lacked both ACS-encoding genes, which are located in different genomic regions. The strain ΔPP_5266 lacked the ACH enzyme, encoded by PP_5266. The two ALD variants were sequentially eliminated to create the double mutant *ΔaldBI ΔaldBII*. Finally, *P. putida* KT2440 *ΔscpC* lacked the AST-based route. All strains were verified for the correctness of genetic modifications through PCR and Sanger sequencing, and then compared to the wild type (Fig. 8). Eliminating each acetate biosynthetic pathway had significant effects on acetate production and overall metabolism. The deletion of the two acetyl-CoA synthase encoding genes (*ΔacsAI ΔacsAII*) reduced the acetate titer by 30% compared to the wild type, while gluconate formation increased substantially at the expense of 2KG production (Fig. 8C and D). Although the peak current reached was similar to that of the wild type, it was achieved 130 h later. Interestingly, unlike the wild type, this mutant exhibited a brief phase of strong acetate production immediately after the process began. The strain lacking acetyl-CoA hydrolase (ΔPP_5266) generated less acetate than the wild type (Fig. 8E). However, it accumulated more gluconate and produced a 20% higher current. In this mutant, gluconate was further oxidized into 2KG only after glucose depletion. The acetate:succinate CoA-transferase-deficient strain (*ΔscpC*) produced 60% less acetate and fully converted glucose into 2KG approximately three days faster than the wild type (Fig. 8F).

**Table 1:** Strains and plasmids

Strains and plasmids	Description	Reference
E. coli		
DH5α λpir	Host for plasmid amplification: *supE44*, *ΔlacU169* (*φ80 lacZΔM15*), *hsdR17* (*rk*^−^*mk*^+^), *recA1*, *endA1*, *thi1*, *gyrA*, *relA*, *λpir* lysogen	Biomedal Life Sci., Seville, Spain
CC118λpir	Mating donor strain: *Δ(ara-leu)*, *araD*, *ΔlacX174*, *galE*, *galK*, *phoA*, *thi1*, *rpsE*, *rpoB*, *argE (Am)*, *recA1*, lysogenic *λpir*	[94]
HB101	Mating helper strain: *SmR*, *hsdR-M*^+^, *pro*, l*eu*, *thi*, *recA*	[95]
Plasmids		
KT2440	Wild type	[2]
*ΔscpC*	KT2440 derivative: *ΔscpC (*PP_0154*)*	This work
ΔPP_5266	KT2440 derivative: ΔPP_5266	This work
*ΔacsAI ΔacsAII*	KT2440 derivative: *ΔacsAI* (PP_4487) *ΔacsAII* (ΔPP_4702)	This work
*ΔaldBI ΔaldBII*	KT2440 derivative: *ΔaldBI* (ΔPP_0545) *ΔaldBII* (or *pedI*, ΔPP_2680)	This work
Plasmids		
pGNW2	Suicide plasmid for integration/deletion: *Km*^*R*^, *oriR6K*,* lacZα* with two flanking I-SceI sites, P 14 g→msfGFP	[74]
pSEVA6213S	Helper plasmid: *Gm*^*R*^, *oriV* (RK2), *xylS*, PEM7→I-SceI	[74]

Notably, *P. putida* KT2440 *ΔaldBI ΔaldBII* exhibited the most significant differences compared to the wild type (Fig. 8G). Acetate production dropped by 80%, indicating that the aldehyde dehydrogenase pathway was the primary route for acetate formation. Remarkably, this mutant demonstrated twice the glucose conversion rate, with complete substrate consumption after just 200 h. During this period, cells converted glucose to 2KG with a yield of 0.96 mol mol^− 1^ glucose, with hardly any gluconate accumulation. From a biotechnological perspective, the ALD mutant outperformed the wild type in terms of yield, selectivity, and productivity for 2KG production. Taken together, transcriptional analysis revealed that all four acetate biosynthesis pathways were upregulated (Fig. 8B). Eliminating any of these pathways resulted in reduced acetate levels (Additional file 2, Figure S5). Lower acetate formation boosted glucose oxidation to 2KG. Additionally, there were notable differences in cell concentration decreases among the strains (Additional file 2, Figure S6).

## Discussion

**Sustained metabolic activity in electrogenic *P. putida *KT2440 through fine-tuned resource allocation.** In this study, we characterized the phenotype of *P. putida* KT2440 cultivated under bio-electrochemical conditions without oxygen (Fig. 1). The strain produced high levels of 2KG and minor amounts of acetate, pyruvate, lactate, succinate, and gluconate (Fig. 1C and D). Remarkably, the non-growing cells remained metabolically active throughout the 16-day process, maintaining their adenylate energy charge (AEC) at a level of 0.52 ± 0.01 (Fig. 2), which is significantly higher than the AEC observed for oxygen-starved *P. putida*, at 0.32 ± 0.01 [34], and 0.28 ± 0.04 [10]. This indicates that the electrogenic metabolism enabled a better energy balance compared to oxygen-limited conditions. However, the ATP content dropped over time (Fig. 2), suggesting that the cells were under stress. This observation aligns with previous findings where *P. putida*, when grown on stress-inducing toxic aromatics, shows a significant decrease in ATP content but is still able to maintain its adenylate energy charge over an extended period [35]. This ability to sustain metabolic activity highlights the fine-tuned resource allocation in *P. putida* KT2440 under electrogenic conditions, enabling prolonged metabolic function and productivity.

The formation of gluconate and 2KG generated electrons that were transferred to the anode in the reactor via the external mediator ferricyanide (Fig. 1). This electron transfer process was crucial for ATP generation [12]. Additionally, the catabolic breakdown of biomass components and the production of acetate contributed to the supply of energy and redox power (Figs. 3 and 4). Various biosynthetic pathways played a role in these processes (Fig. 8). Among these pathways, the acetate:succinate CoA-transferase (AST) and reverse acetyl-CoA synthase (ACS) pathways were directly associated with ATP formation, while the aldehyde dehydrogenase (ALD) pathway generated NAD(P)H. When these routes were deleted, *P. putida* reinforced its primary ATP synthesis pathway,  periplasmic glucose oxidation (Fig. 8). This adaptive response is similar to that observed in other microbes, which enhance their ATP supply mechanisms under energy-limiting conditions [36].

In addition, *P. putida* KT2440 drastically reduced its energy consumption, likely in response to the lower ATP content observed (Fig. 2). Multi-omics data revealed that this adaptation was facilitated by global resource allocation at the levels of transcriptional expression (Fig. 5), protein abundance (Figs. 5 and 6), and metabolite concentration (Figs. 3 and 4). The major adaptations occurred within the first 24 h. Notably, there was a significant downregulation of the ATP-intensive flagellar motor (Additional file 2, Table S4) and the silencing of the translation apparatus, particularly during the initial 100 h (Figs. 5, 6 and 7). This involved the energy-dependent translational throttle protein EttA (Fig. 6), which restricts translation based on reduced energy levels (Fig. 2) [37]. Under optimal growth conditions, up to 80% of anabolically-required ATP is consumed for protein and rRNA synthesis [38, 39]. Therefore, the reduction in overall translation output was crucial for the sustained performance of *P. putida* in the BES over an extended period. Obviously, the cells entered a new state of homeostasis, where existing proteins were maintained and remained active with low energy requirements. To support the maintenance of cellular protein homeostasis, the abundance of major ATP-dependent chaperones GroEL/S, DnaK, and the ATP-independent chaperone Trigger Factor remained unchanged (Additional file 3, Table S1).

Under unfavorable conditions, bacteria typically stabilize ribosomes in two forms: as 70 S particles, with Factor pY bound to the small subunit, or as 100 S particles, a complex of two 70 S units coupled together by the Hibernation Promoting Factor (HPF) and the Ribosome Modulation Factor (RMF) [40, 41]. However, in this study, hibernating ribosome complexes were not formed in *P. putida* KT2440. A search of our datasets revealed no gene copy encoding Factor pY in the genome, and HPF was strongly downregulated at both the transcript and protein levels, explaining the absence of 70 S and 100 S particles in the sucrose gradients (Fig. 7). In contrast, *Pseudomonas aeruginosa*, a pathogenic relative which can grow anaerobically in the presence of nitrate and nitrite, induces ribosome hibernation under anaerobic conditions [42]. This suggests that anoxic-electrogenic *P. putida* may prefer to degrade and/or recycle proteins and ribonucleic acids for catabolic processes rather than maintaining a hibernating ribosome pool, in line with later phase growth arrest [43]. Such degradation processes would result in the loss of nitrogen, consistent with the increased C: N ratio observed during the process (Fig. 4A). As shown, the adaptation to electrogenic conditions in *P. putida* KT2440 was marked by the accumulation and subsequent re-consumption of extracellular organic acids, ranging from two to four carbons (Fig. 1D). The exact reasons for the complex dynamics observed, such as the initial export of succinate or the re-consumption of lactate and pyruvate, remain unclear based on the current data. However, organic acid accumulation in *P. putida* has been previously noted under various conditions and appears to be a natural response to specific culture environments. For instance, citrate accumulation has been observed during polyhydroxyalkanoate (PHA) production from glycerol while succinate and malate accumulation occurred during PHA production in continuous culture [44]. Additionally, pyruvate and acetate accumulation has been linked to the stringent response to (p)ppGpp accumulation in *P. putida* [21]. To gain a deeper understanding of these dynamics, it would be beneficial to study the organic acid profiles during the initial adaptation of *P. putida* in the BES with shorter sampling intervals and greater detail. This approach could provide more insights into the mechanisms driving organic acid accumulation and re-consumption, enhancing our understanding of the microbe’s adaptive strategies under electrogenic conditions.

**Acetyl-CoA serves as a central hub involved in the supply of energy and redox power.** In addition to the rearrangement of protein synthesis, pronounced changes were observed in fatty acid metabolism. The ^13^C labelling pattern of acetate in the tracer experiment indicated a strong incorporation of biomass-derived carbon (^12^C) (Fig. 3A). In consequence, acetate was not exclusively formed from glucose, as initially assumed. This discovery impacted the carbon and energy balances, which appeared overestimated based on stoichiometry analysis alone. Assuming the SFL of acetate remained consistent at the end of the process, with only 39.4% of acetate originating from glucose, the electron balance was recalculated to be 98.2 ± 2.4%. Similarly, the carbon balance, was adjusted to 96.9% ± 2.4%. While this estimate is based on the ^13^C labelling pattern of acetate after 100 h (and not at the end of the process, where no sample was taken) and should be therefore interpreted cautiously, both balances closed more accurately. Incorporating ^13^C-tracer information therefore proves useful, as it allows to validate the origin of products.

Based on the elevated acetyl-CoA pool and the reduction in cellular fatty acid content over time, we conclude that acetate was at least partially derived from lipid degradation (Fig. 4C and D). This conclusion aligns with observations in various microbes, which break down lipids for energy and redox power during stationary phase [45–47]. Considering an average fatty acid carbon chain length of 16 (Additional file 2, Table S2), the degradation of one such molecule yields 7 FADH, 7 NADH, and 8 acetyl-CoA. The acetyl-CoA generated evidently contributed to acetate production (Figs. 1D and 3A), providing additional ATP (Fig. 8). We propose that fatty acid degradation served as an additional energy and reducing power source for electrogenic *P. putida*. The importance of these catabolic reactions is supported by their transcriptional activation. For instance, genes involved in β-oxidation (*fadA*,* paaF*,* paaH*,* pcaF-I*) were upregulated (Additional file 2, Table S5). Concurrently, genes encoding enzymes for de novo fatty acid synthesis were downregulated at both the transcript (*mmgF*,* prpC*,* accA*,* accB*,* atoB*,* fabA*) and protein levels (*fabD*,* acpP*) (Additional file 2, Table S5).

Notably, *fabA* and *fabD* are involved in the synthesis of unsaturated fatty acids. We observed an increase in the saturation degree of the cellular fatty acid pool (Additional file 2, Table S2), which could serve as a mechanism to adjust membrane fluidity [48, 49]. Higher saturation might also act as an additional electron sink. The absence of odd-chain fatty acids explained the downregulation of genes associated with the methyl-citrate cycle (Additional file 2, Table S5) [19, 50]. Our data, along with transcriptional changes in central carbon metabolism (Fig. 5C), highlight the pivotal role of acetyl-CoA in the metabolic network of electrogenic *P. putida*. Our labelling experiments suggested the involvement of acetyl-CoA in exchange reactions between the EMP pathway and the TCA cycle (Fig. 3A) which affects ^13^C metabolite labelling [51]. These reactions have been previously observed in *P. putida* KT2440 and *P. aeruginosa* PAO-1 during aerobic growth on glucose [52, 53].

Furthermore, the metabolism of fatty acids was adapted for structural reasons, notably marked by a significant increase in trans-unsaturated fatty acids (Additional file 2, Table S2). Given the shutdown of fatty acid biosynthesis (Additional file 2, Table S5), these trans-unsaturated fatty acids were likely not synthesized de novo. Based on the observed fatty acid spectrum, we conclude that they originated from the cis-trans isomerization of existing unsaturated fatty acids. This conclusion is supported by the strong upregulation of the non-reversible cis-trans isomerase Cti (Additional file 2, Table S5). Consequently, the overall cis-trans fatty acid ratio shifted dramatically from 25.1 at the process start to 0.6 after 100 h, indicating that most of the cis-oriented fatty acids were converted (Additional file 2, Table S2). This type of adaptation has been shown to occur in *P. putida* strains (P8, NCTC 10936, and KT2440) when subjected to abrupt disturbances without the ability to synthesize new fatty acids [54]. This was apparently the case here. The isomerization process, which occurred without altering the double bond position and without requiring a cofactor or energy, provided an efficient mechanism for adjusting the membrane composition [29, 55, 56]. The resulting membrane composition had higher rigidity and tighter packing with reduced fluidity [57] and enhanced cellular resilience. It should be noted that the altered membrane composition probably supported the maintenance of efficient long-term function of the involved electron transport chain complexes, as well as the transporters for substrates, products, and the mediator.

**The elimination of acetate biosynthetic pathways leads to metabolically engineered mutants with superior 2KG production.** Three mutants - *ΔaldBI ΔaldBII*,* ΔscpC*, and ΔPP_5266 - exhibited increased 2KG production, underscoring the elimination of acetate formation as a key metabolic engineering strategy for enhancing product formation in electrogenic *P. putida*. This marks the first successful metabolic engineering strategy outside of the 2KG biosynthetic pathway itself. Among these, the *ΔaldBI ΔaldBII* mutant demonstrated the best performance (Fig. 8). It outperformed the wild type in titer, yield, and productivity, forming 2KG almost twice as fast and achieving a substantially increased yield of 0.96 mol mol^− 1^ glucose. This mutant produced minimal amounts of gluconate and five times less acetate than the wild type. Despite the clear phenotype, the exact pathway components involved in acetate formation remain unclear. Based on our data, we hypothesize that the pathway starts from acetyl-CoA and proceeds via acetaldehyde to acetate, with the latter step involving the two aldehyde dehydrogenases (AldBI/II). However, no gene encoding an acylating acetaldehyde dehydrogenase (acetyl-CoA to acetaldehyde) was found in the *P. putida* KT2440 genome. Possible alternative sources of acetaldehyde include threonine (degradation via *ltaE* and PP_0488) and phospholipid phosphatidylethanolamine (degradation via *eutB* and *eutC*) (Fig. 8B). Threonine degradation was transcriptionally upregulated (log2 fold change 1.0 for ltaE and 2.6 for PP_0488), likely activated as part of protein and amino acid catabolism for resource allocation. Conversely, phosphatidylethanolamine degradation was downregulated (log2 fold change *eutB*: -3.02; *eutC*: -3.08). However, these alternative sources did not appear to be available in sufficient quantities to account for the high amount of unlabelled acetate (1.8 mM) (Fig. 3A). Unlike in other microbes where acetate is a well-studied overflow metabolite [36, 58], acetate formation in *P. putida* KT2440 is less understood, likely due to its lack of importance under common aerobic growth conditions. Further research is needed to elucidate the complete picture of acetate biosynthesis in this strain.

As stated above, the naturally aerobic metabolism of *Pseudomonas putida* poses challenges for its industrial use. Aerobic processes typically increase capital costs, limit reactor size, and result in significant carbon loss via CO_2_ formation, leading to lower yields compared to anaerobic conditions [12, 59]. Therefore, the newly engineered 2KG-producing mutants of *P. putida*, optimized for anoxic-electrogenic production, present an attractive alternative. 2KG is a commercially valuable chemical with diverse applications, similar to other organic acids [60–62]. It is used in metal complexes for antitumor treatments [63] and as a bioactive cosmetic ingredient to boost hyaluronic acid production, enhancing skin rejuvenation and elasticity [64]. According to the International Nomenclature of Cosmetic Ingredients (INCI), 2KG is included in more than 20 commercial products. Additionally, 2KG is industrially utilized to synthesize isoascorbic acid, an antioxidant approved for food preservation in the European market [65].

The new 2KG-producing mutants can be utilized in various operational modes, including batch processes with high initial glucose levels, fed-batch processes with continuous glucose feeding, or a two-stage process: an initial aerobic phase for biomass formation followed by an anaerobic phase for non-growth 2KG production. This approach could result in significant overall yields (Fig. 8) and long-term operation (Fig. 1B). *P. putida* also efficiently oxidizes other sugars like fructose under bio-electrochemical conditions, achieving high yields [15]. Even non-growth supporting carbohydrates can be converted into valuable sugar acids. For example, arabinose and galactose can be converted into L-arabonic and L-galactonic acid, respectively, both of which are commercially attractive chemicals [66, 67]. Moreover, the product spectrum can be expanded by using carbon sources other than sugars. For instance, citrate can be oxidized into *p*-hydroxybenzoate using recombinant *P. putida* KT2440 in a BES [68]. It appears interesting to test, if the synthesis of these interesting products is also enhanced in *P. putida* Δ*aldBI* Δ*aldBII*. At present, however, the achieved performance seems far from industrial efficiency, requiring further rounds of optimization. Hereby, the combined deletion of *aldBI*,* aldBII* and Δ*scpC* appears promising to further reduce or even completely abolish acetate formation and improve formation of the target products.

## Conclusions

This work delivers the first comprehensive systems biology insight into the electrogenic phenotype of *P. putida* KT2440 through carefully conducted transcriptomic, proteomic, and metabolomic analyses. Major adaptation mechanisms of the microbe to anoxic-electrogenic conditions were unravelled, including the activation of protein and lipid catabolism, acetate formation, and formation of gluconate and 2KG to derive energy and reducing power. Additionally, cells conserved energy by shutting down translation and mobility. These adaptations, embedded within major rearrangements of central carbon metabolism, enabled the cells to maintain stable energy levels and substantial metabolic activity for over two weeks. Inspired by these findings, mutants affected in acetate production were created, demonstrating superior 2KG production in terms of titer, yield, and productivity compared to the wild type. The periplasmic 2KG route was enhanced to compensate for the reduced energy supply due to attenuated acetate production, making these mutants attractive for enhanced 2KG production under anoxic-electrogenic conditions. Future optimization could involve eliminating multiple acetate pathways to further enhance performance. The best 2KG-producing mutant reached a plateau in current output while the mediator pool turned largely reduced, suggesting other bottlenecks in the electrogenic process, such as bioreactor design or mediator turnover, which need to be addressed (Additional file 2, Fig. S7).

**Fig. 9:**
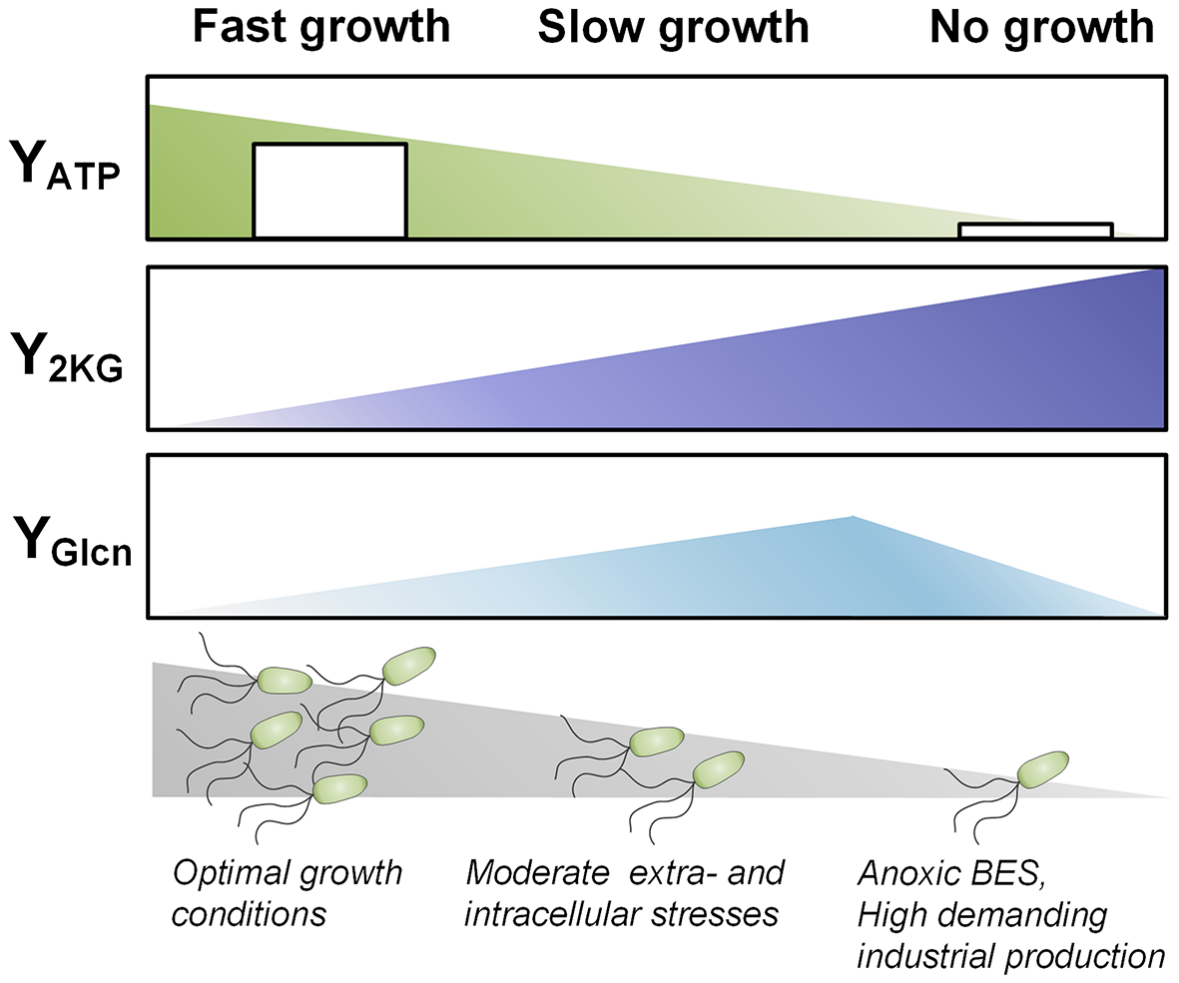
Overall impact of the growth mode on carbon and energy metabolism. The scheme highlights the interplay between the 2KG yield, the ATP yields and the growth status for fast growth, slow growth, and no growth

The non-growth anoxic-electrogenic mode extends our understanding of the interplay between glucose phosphorylation and glucose oxidation into gluconate and 2KG [69] (Fig. 9). Under balanced growth conditions, only minor amounts of glucose are fully oxidized to 2KG, with about 90% of the carbon flux bypassing this node. In contrast, stressed and nutrient-limited, slowly growing cells secrete elevated levels of gluconate and 2KG [70, 71]. In the anoxic-electrogenic mode, cells almost exclusively convert glucose into 2KG, achieving up to 96% conversion (as seen in *P. putida ΔaldBI ΔaldBII*). This exclusive 2KG formation at zero growth was also observed during later stages of demanding fed-batch processes on lignin-based aromatics [72], indicating that this response is the ultimate mode when growth is no longer possible. The periplasmic glucose oxidation route allows cells to partially uncouple ATP formation from NADH formation [12], balancing the trade-off between protein investment for glucose mineralization and energy yield. Taken together, these insights pave the way for optimizing *P. putida* KT2440 for industrial bio-electrochemical applications, highlighting the potential of non-growth anoxic-electrogenic conditions to maximize product yield and efficiency.

## Materials and methods

**Bacterial strains.***P. putida* KT2440 (DSM 6125) was obtained from the German Collection of Microorganisms and Cell Cultures (Leibniz-Institute DSMZ - Deutsche Sammlung von Mikroorganismen und Zellkulturen GmbH, Braunschweig, Germany). *E. coli* DH5α λpir (Biomedal Life Science, Seville, Spain) and *E. coli* CC118λpir [35] were used for cloning purposes. All strains are listed in Table 1.

**Genetic engineering.** Genomic gene deletions were done based on chromosomal integration of a suicide plasmid (pGNW2) followed by the action of the homing endonuclease I-SceI to remove the plasmid backbone [73]. In short, the linearized integrative plasmids and 500 bp-fragments, reflecting the upstream and downstream flanking regions of genes to be deleted were assembled in vitro. The assembled plasmids were verified by PCR and delivered to *P. putida* via tri-parental mating. Obtained *P. putida*::pGNW2-UP/DW clones were transformed with the plasmid pSEVA6213S (bearing I-*SceI*) for counter-selection. The primers used for genetic engineering can be found in the supplementary (Additional file 2, Table S1). All deletions were verified by PCR and Sanger sequencing (GENEWIZ Azenta Life Sciences, Leipzig, Germany).

**Set-up and operation of the bio-electrochemical system (BES).** Defined M9 glucose medium (DM9) [12] was used for the pre-culture (5 g L^− 1^ glucose) and the bio-electrochemical cultivation (1.5 g L^− 1^ glucose). For the latter, the medium was amended with ferricyanide, i.e. [Fe(CN)_6_], as the mediator. For pre-culture preparation, a single colony from a fresh LB plate was used to inoculate a baffled shake flask filled with DM9 medium (< 15% of total volume), incubated overnight (16 h). Cells were then harvested by centrifugation (5,000 x *g*, RT, 5 min) and used to inoculate the bio-electrochemical reactor to an initial OD_600_ of approximately 1. The exact cell concentration was measured at the start. Reactor configuration and operation were as previously described [74] (Fig. 1A). The potential was set to 0.5 V versus the reference electrode (013691 Ag/AgCl electrode in saturated KCl, RE-1CP, ALS, Tokyo, Japan). For isotopic tracer studies, glucose was replaced with 99% [^13^C_6_] glucose ([U-^13^C] glucose, CLM-1396-10, Cambridge Isotope Laboratories, Tewksbury, MA, USA). The set-up exhibited a slight evaporation of water (0.09 mL h^− 1^). This value was used to correct the measured levels of products and substrates for the resulting concentration effects prior to the calculation of rates and yields [75] (Additional file 1). For systems biology analysis, samples taken from the inoculum at the process start, designated start sample, were compared to samples taken from the bio-electrochemical process at different time points.

**Calculation of electron and carbon balance.** The electron balance (EB) was determined using Eq. 1:

1 $$\eqalign{& EB{\bf{\it{ }}}\left[ {\bf{\it{\% }}} \right] = \left( {\sum\nolimits_{i = n}^n {\left( {{C_i}_{{t_{end}}}*{m_i}*{\rm{\gamma }}} \right) + {e_{anode}}_{{t_{end}}})} } \right. \cr & \left. {/(\mathop \sum \limits_{i = n}^n \left( {{C_i}_{{t_0}}*{m_i}*{\rm{\gamma }}} \right) + {e_{anode}}_{{t_0}}} \right){\bf{\it{*}}}100{\rm{ }} \cr}$$

where C_i_ displays the quantity of each compound at the start point (t_0_) and end point of the process (t_end_), and m denotes the corresponding number of carbon atoms in a molecule. γ denotes the degree of reduction of the respective compounds, and e_anode_ is the quantity of electrons collected at the anode at the start of the process (t_0_) and end point of the process (t_end_).

The carbon recovery (CR) was determined using Eq. 2:

2 $$CR{\bf{\it{ }}}\left[ {\bf{\it{\% }}} \right] = \sum\nolimits_{i = n}^n {\left( {{C_i}_{{t_{end}}}*{m_i}} \right)/\mathop \sum \limits_{i = n}^n \left( {{C_i}_{{t_0}}*{m_i}} \right){\bf{\it{*}}}100}$$

where C_i_ displays the quantity of each compound at the start point (t_0_) and end point of the process (t_end_), and m denotes the corresponding number of carbon atoms in a molecule.

**Quantification of cells**,** substrates**,** and products.** The cell concentration was analysed as optical density (OD_600_) by a spectrophotometer at 600 nm. The concentration of ferricyanide ([Fe(CN)_6_]^3−^) was spectrophotometrically determined at 420 nm [12]. The concentration of glucose was quantified by HPLC (1260 Infinity Series, Agilent, Darmstadt, Germany) using a MetaCarb 87C column (Agilent) at 80 °C as the stationary phase, and deionized water at a flow rate of 1 mL min^− 1^ as the mobile phase. Glucose was detected via the refractive index, and external standards were used for quantification. Organic acids (gluconate, 2KG, pyruvate, acetate, succinate, lactate) were quantified by HPLC (1260 Infinity Series, Agilent) using an Aminex HPX-87H column (Bio–Rad, Hercules, CA, USA) at 40 °C as the stationary phase, and isocratic elution with 50 mM H_2_SO_4_ at a flow rate of 0.5 mL min^− 1^ as the mobile phase. The analytes were detected by UV absorption at 210 nm quantified via external standards.

**Elemental composition of biomass.** The CHN content of freeze-dried biomass (*n* = 3) was determined by elemental analysis (Elementar Vario MICRO, Elementar Analysensysteme GmbH, Langenselbold, Germany).

**Quantification of adenylate energy metabolites.** A sample (5 mg cell dry weight) was harvested from the cultivation via vacuum filtration (Durapore Membrane, PVDF, 0.45 μm, 47 mm, Millipore Merck, Darmstadt, Germany) [76]. The filter was submerged in 4 mL boiling ethanol/water (75:25 v/v, 70 °C). The mixture was vortexed for 30 s, and then centrifuged (17,000 × *g* 4 °C, 5 min). Cell debris was discarded. The supernatant was transferred into pre-cooled 50 mL falcons, diluted with ice-cold water to an ethanol content of 20% (v/v), frozen at − 80 °C, and lyophilized. The lyophilizate was resuspended in 0.5 mL ATP assay buffer (ab113849, Abcam, Cambridge, UK). Protein was removed (10 kDa, Vivaspin 500, GE Healthcare, Freiburg, Germany). The ATP content in the obtained extract was quantified according to the manufacturer’s protocol (ab113849 Kit, Abcam) using a GloMax microplate reader (Promega, Madison, WI, US). Prior to analysis, ADP and AMP were enzymatically converted to ATP. For conversion of ADP to ATP, 100 µL extract was incubated in 25 µL reaction buffer (75 mM potassium phosphate (pH 7.3), 15 mM MgCI_2_, 0.5 mM phosphoenolpyruvate, and 36 mg mL^− 1^ pyruvate kinase (P9136, Sigma-Aldrich)). For conversion of AMP to ATP, 100 µL extract was incubated in 25 µL reaction buffer, additionally supplemented with 4 mg mL^− 1^ myokinase (M5520, Sigma-Aldrich). Each reaction mixture was incubated for 15 min at 37 °C. Subsequently, the enzymes were deactivated (100 °C, 2 min).

Based on the data, the adenylate energy charge (AEC) [77] was calculated using Eq. 3:

3 $$\:\text{A}\text{E}\text{C}\:\left[-\right]=\:\frac{\left[\text{A}\text{T}\text{P}\right]+0.5\text{*}\left[\text{A}\text{D}\text{P}\right]}{\left[\text{A}\text{T}\text{P}\right]+\left[\text{A}\text{D}\text{P}\right]+\left[\text{A}\text{M}\text{P}\right]}$$

**Extraction and quantification of fatty acids.** The analysis of cellular fatty acids was carried out by DSMZ Services (Leibniz-Institute DSMZ -Deutsche Sammlung von Mikroorganismen und Zellkulturen GmbH, Braunschweig, Germany).

**Total fatty acid content**. The content of fatty acids in the biomass was quantified as previously described [78]. In short, a cell sample (5 mg cell dry weight) was freeze-dried, transferred into a glass vial, and amended with 300 µL of a mixture of methanol, toluene, and 95% sulfuric acid (50:50:2 v/v/v) for extraction and transesterification. Afterwards, 15 µg n-3 heneicosapentaenoic acid methyl ester (HPA, 22:5, Cayman-Chemical, Ann Arbor, MI, USA) was added as internal standard. The mixture was incubated at 80 °C for 24 h. After reaching room temperature, 250 µL stopping solution (0.5 M NH_4_HCO_3_ and 2 M KCl in H_2_O) was added, and the mixture was centrifuged (12,000 x *g*, RT, 5 min). The upper, organic phase was used for GC/MS analysis.

**Extraction and quantification of intracellular CoA thioesters.** The analysis of intracellular CoA thioesters was done as previously described [79]. Shortly, 8 mg cells were harvested, transferred into cooled extraction and quenching buffer (95% acetonitrile, 25 mM formic acid, -20 °C), and incubated on ice for 10 min. Afterwards, the mixture was centrifuged (15,000 × *g*, 4 °C, 10 min), and the supernatant was mixed with 10 mL super-cooled deionized water. The pellet was resuspended in 8 mL super-cooled water and centrifuged again. The obtained supernatant was combined, snap-frozen in liquid nitrogen, freeze-dried, re-dissolved in 500 µL cooled resuspension buffer (25 mM ammonium formate, pH 3.0, 2% MeOH, 4 °C), and filtered (Ultrafree-MC 0.22 μm, Merck, Millipore, Germany). Analysis was then done using a triple quadrupole MS (QTRAP 6500+, AB Sciex, Darmstadt, Germany), coupled to an HPLC system (Agilent Infinity 1290 System), equipped with a core-shell reversed-phase column (Kinetex XB-C18, 100 × 2.1 mm, 2.6 μm, 100 Å, Phenomenex) at 40 °C. For analysis, 10 µL sample was injected and separated using a gradient of formic acid (50 mM, pH 8.1 with ammonium hydroxide 25% in H_2_O, A), and methanol (B) at a flow rate of 300 µL min^− 1^. The gradient was as follows: 0–7 min, 0–10% B; 7–10 min, 10–100% B; 10–11 min, 100% B; 11–12 min, 100–0% B; 12–15 min, 0% B.

**GC-MS**^**13**^**C labelling analysis of amino acids.** Protein-bound amino acids were analyzed using 2 mg CDW, hydrolyzed by incubation in 100 µL 6 M HCl at 100 °C for 24 h. Subsequently, cell debris was removed by filtration (0.2 μm, Ultrafree-MC, Merck-Millipore, Darmstadt, Germany). The obtained hydrolysate was dried under a nitrogen stream, resuspended in 50 µL N, N-dimethylformamide containing 1% (v/v) pyridine, and derivatized with 50 µL N-methyl-*t*-butyldimethylsilyl-trifluoroacetamide (MBDSTFA, Macherey-Nagel, Düren, Germany) at 80 °C for 30 min [80].

For ^13^C labelling analysis of free intracellular amino acids, 4 mL fermentation sample was harvested by vacuum fast filtration (cellulose nitrate membrane filter, 0.2 μm, 47 mm, Sartorius) followed by hot water extraction [81]. The filter was washed once with 15 mL NaCl solution, matching the ionic strength of the medium, and placed in a plastic cup. After incubation at 100 °C (10 min), the obtained extract was cooled on ice and centrifuged (13,000 x *g*, 4 °C, 5 min). For analysis, 1 mL supernatant was vacuum-dried and derivatized in the same way as samples for protein-bound amino acids. The mass isotopomer distributions (MIDs) of the derivatized amino acids were analyzed by GC-MS (Agilent 7890A, Quadrupole Mass Selective Detector 5975C, Agilent Technologies), equipped with an HP-5MS column (30 m, 250 μm x 0.25 μm, Agilent Technologies) using helium 5.0 as the carrier gas (1.7 mL min^-1^). The temperature program was as follows: 120 °C (0–2 min), 8 °C min^− 1^ increase (2–12 min), 10 °C min^− 1^ increase (12–24.5 min), and 325 °C (24.5–27 min). Further settings controlled the inlet (250 °C), the transfer liner (280 °C), the ion source (230 °C), and the quadrupole temperature (150 °C) respectively. Selective ion monitoring (SIM) was used to quantify the MIDs of amino acid fragments with appropriate quality, i.e. alanine (*m/z* 260), valine (m/z 288), leucine (*m/z* 274), threonine (*m/z* 404), aspartate (*m/z* 418), serine (*m/z* 390), and proline (*m/z* 258) [82].

**GC–MS and LC-MS/MS**^**13**^**C labelling analysis of organic acids.** The MIDs of fragment ions of organic acids – acetate (*m/z* 43), pyruvate (*m/z* 174), and succinate (*m/z* 289) were analyzed by GC-MS using an HP-5MS column (Agilent) as the stationary phase and helium 5.0 as the mobile phase. For the analysis of acetate, 100 µL supernatant was mixed with 100 µL H_2_SO_4_ (10% vol/vol) and 20 µL *n*-pentanol, respectively, and incubated at 80 °C for 15 min [83]. The mixture was cooled down on ice, and the formed ester was extracted with 200 µL *n*-hexane. The following temperature program was used for GC/MS-analysis: 75 °C for 2 min, ramp: 25 °C min^− 1^ For the analysis of pyruvate, 200 µL of supernatant was dried under a nitrogen stream. The dried sample was dissolved in 50 µL methoxamine hydrochloride in pyridine (20 mg mL^-1^) and incubated at 80 °C for 30 min. Subsequently, 50 µL MSTFA (Macherey-Nagel, Düren, Germany) were added, and the mixture was incubated at 80 °C for 30 min. The following temperature program was used for analysis: 30 °C (0–1 min), 10 °C min^− 1^ increase (1–10 min), and 40 °C min^− 1^ increase (10–15 min). For the analysis of succinate, 50 µL of supernatant was dried under nitrogen stream. Derivatization and instrument settings were the same as for amino acid measurement. The labelling patterns of 2KG and gluconate were analyzed as fragments ions by LC-MS/MS, using a triple quadrupole MS (QTRAP 6500+, AB Sciex, Darmstadt, Germany), coupled to an HPLC system (Agilent Infinity 1290 System), equipped with a Acquity UPLC BEH Amide column (100 × 2.1 mm, 1.7 μm, 130 Å, Waters) at 40 °C. For analysis, 10 µL sample was injected and separated using a gradient of NH₄OH (0.1%, pH 8.9, A), and ACN (B) at a flow rate of 200 µL min^− 1^. The gradient was as follows: 0–7 min, 100% B; 7–10 min, 100 − 70% B; 10–14 min, 70 − 50% B; 15 min, 50–100% B.

All obtained ^13^C enrichments were corrected for natural abundance of isotopes [84], and presented as summed fractional labelling (SFL) [85]. Naturally labelled substances exhibited a SFL of 0.02 ± 0.02%.

**Global gene expression analysis.** A customized microarray (eArray, SurePrint G3, 8 × 60 K, Agilent Technologies) based on the recently revisited genome sequence of *P. putida* KT2440 [86] was manufactured. The microarray consisted of three different 60-mer probes per gene as well as internal controls [87]. For sampling, 2 mL of sample was quickly centrifuged (13,000 rpm, 30 s). The supernatant was discarded, and the cell pellet was snap-frozen in liquid N_2_. Total RNA was isolated and purified with the RNeasy Mini Kit (Qiagen, Hilden, Germany), and TURBO DNA-free Kit (Thermo Fisher Scientific, Waltham, MA, USA) and subsequently quantified (NanoDrop 1000, Peqlab Biotechnology, Erlangen, Germany). The RNA quality was evaluated using a Bioanalyzer System (RNA 6000 Nano Kit, 2100 Bioanalyzer System, Agilent Technologies). Only samples with an RNA integrity number (RIN) > 8 were used for further processing. Afterwards, 50 ng RNA was used as input for direct chemical labelling following the manufacturer’s protocol (Low Input Quick Amp WT Labeling One-Color Kit, RNA Spike-In One-Color Kit, Agilent Technologies). Then, labelled RNA was purified (RNeasy Mini Spin Columns, Agilent Technologies), and the cRNA was quantified (NanoDrop 1000, Peqlab, Erlangen, Germany). Here, it was ensured that at least 600 ng of cRNA with a specific activity of 15 pmol Cy3 µg^− 1^ cRNA were used for hybridization onto the microarray [88]. Hybridization was done according to the manufacturer’s protocol (Gene Expression Hybridization Kit, SureHyb chamber Agilent Technologies). Thereafter, the microarray was washed (Gene Expression Wash Buffer Kit, Agilent Technologies), transferred to the SureScan microarray scanner cassette (G2600D, Agilent Technologies), and scanned (SureScan Microarray Scanner G4900DA, Agilent Technologies) using the AgilentG3_GX_1color scanner protocol with 3 μm double resolution. Data extraction and processing were conducted using microarray and feature extraction software (Version 12.1.1.1, Agilent Technologies). Transcriptomic data analysis and visualization were done using GeneSpring software (Version 14.9, Agilent Technologies) and Perseus Version 1.6.15 [89]. For statistical analysis, an unpaired t-test was applied with asymptotic computation of p-values adjusted for multiple testing according to the Benjamini-Hochberg method [90]. The q-value cut-off was set to 0.05. The data were then filtered for genes with a log_2_fold change ≥ 2 (p-value ≤ 0.05). The entire data set can be accessed at GEO (GSE266590).

**Ribosome analysis.** For ribosome profiling, cells (10 mg cell dry weight) were harvested in a 50 mL falcon tube filled with 25 g ice and 50 µL chloramphenicol (100 mg mL^− 1^). Afterwards, the sample was centrifuged (5,000 x *g*, 4 °C, 5 min). The cell pellet was resuspended on ice in 1 mL buffer (50 mM Tris pH 8, 100 mM NaCl, 10 mM MgCl_2_, 100 µg mL ^− 1^ chloramphenicol, 1 mM DTT), transferred to a 2 mL tube, and centrifuged (5,000 x *g*, 4 °C, 5 min). The supernatant was discarded, and the pellet was resuspended in 250 µL of the same buffer. The suspension was directly pipetted in liquid nitrogen. The frozen drops were collected in 2 mL tubes and stored at -80 °C. Prior to analysis, three samples were merged and resuspended in 250 µL lysis buffer (50 mM Tris pH 8, 100 mM NaCl, 10 mM MgCl_2,_ 100 µg mL^− 1^ chloramphenicol, 1 mM DTT, 0.75 mg mL^− 1^ lysozyme (L-6876, Sigma Aldrich, Taufkirchen, Germany), 1% [w/v] TRITON X-100, 10 mM Ribonucleoside Vanadyl Complex (S1402S, NEB, Frankfurt am Main, Germany), 1% [v/v] Protease Inhibitor Cocktail (P8849-1ML, Sigma Aldrich, Taufkirchen, Germany), lysed in two cycles at 6,500 rpm (Precellys 24, Bertin Technology, France) each 30 s with 1 min on ice in between, and subsequently centrifuged (13,000 rpm, 4 °C, 10 min) to remove cell debris. Then, 500 µL extract was transferred to a tube pre-filled with a sucrose gradient (10–50%), followed by ultracentrifugation (Optima LE-80 K, Beckman Coulter, United States, 34,500 x *g*, 4 °C, 2 h). The absorbance readout of the samples was monitored at 260 nm at a pump rate of 1 mL min^− 1^.

**Proteome analysis.** For analysis of the total proteome, a shotgun approach was applied. In short, 2 mL samples were centrifuged (13,000 x *g*, 4 °C, 1 min). The obtained cell pellets were immediately frozen in liquid nitrogen. Disruption of the cells was carried out by freeze-thaw cycles. For this purpose, the pellets were dissolved in 50 µL of 50 mM ammonium bicarbonate buffer, frozen in liquid nitrogen, and then incubated for 60 s at 40 °C and 750 rpm. This cycle was repeated three times. After the last round, samples were placed in an ultrasonic bath for 30 s. The protein concentration was measured (2D-Quant kit, Cytiva, Marlborough, MA USA), and 10 µg of protein was further processed. To this end, 0.04 µg glyceraldehyde 3-phosphate dehydrogenase (*Staphylococcus aureus* Mrsa252, 336 aa residues) was added as internal standard. Then, 2 µL of 1 M dithiothreitol was added, and samples were incubated in a thermomixer (30 °C, 1 h, 400 rpm). Afterwards, 15 µL of 400 mM 2-iodacetamine was added, and the mixture was incubated at RT and 400 rpm for 1 h in the dark. Digestion of proteins to peptides was initiated by the addition of 0.63 µg trypsin (from porcine, sequencing grade, Promega), followed by incubation overnight at 37 °C and 400 rpm. The digestion reaction was stopped by adding 1 µL of 100% formic acid. Finally, samples were desalted using a ZipTip-µC18 column (Merck Millipore, Darmstadt, Germany), and lyophilized. The dried samples were resuspended in 0.1% formic acid and analyzed via nano-liquid chromatography (Dionex Ultimate 3000RSLC, Thermo Scientific, USA) coupled to an Orbitrap Fusion Trihybrid tandem mass spectrometer (MS/MS; Thermo Scientific, USA). LC-MS/MS parameters were chosen according to previous work [91]. Data were statistically analyzed in Perseus Version 1.6.15. First data were log*2* transformed and contaminants were filtered out. Proteins were removed that had less than three valid values in each condition. After normalization by mean subtraction, samples were compared by a two-sided unpaired t-test using permutation-based FDR calculation (S0 = 0.1) to assess, if in both groups the protein was quantified in at least three replicates, respectively. Only differences, exhibiting a p-value and q-value lower than 0.05, and a log_2_-fold-change of <-1 or > 1, respectively, were regarded significant. Significantly changed proteins were divided into groups, based on their gene ontology “biological process” annotation, which was retrieved from the Perseus annotation database. The fold-change of entities in the groups was subjected to hierarchical clustering. The entire data set can be accessed at MassIVE (MSV000094887).

**Data processing and statistical analysis.** Results are shown as mean values and corresponding standard deviations. Statistical analysis was conducted by Student’s t-test or one-way analysis of variance (ANOVA). Means were considered significantly different, if the p-value was < 0.05 (*), and < 0.01 (**), respectively. Statistical analysis was performed using originLab (originPro 2023b), Perseus (Version 1.6.15), and GeneSpring software (Version 14.9).

## Electronic supplementary material

Below is the link to the electronic supplementary material.

Supplementary Material 1: Additional file 1: Figure S1. BES cultivation of *P.putida* KT2440 (Fig. 1).The displayed data were corrected for the evaporation of water. Table S1: Mean values of measured data (uncorrected) and data corrected for the evaporation of water (corrected). (*n* = 4).

Supplementary Material 2: Additional file 2: Table S1-List of primers used for genetic engineering. The overhangs for Gibson assembly are underscored. Table S2: Fatty acid composition of *P. putida* KT2440 at the start of the process (0 h) and after 100 h incubation in the bio-electrochemical system. The data are given in % of total fatty acids. Table S3: Impact of anoxic-electrochemical conditions on the expression of genes related to central carbon metabolism in *P. putida* KT2440. The data reflect significant differences between process start (0 h) and 24 h incubation in the bio-electrochemical system. *n* = 3. Table S4: Impact of anoxic-electrochemical conditions on the expression of genes related to assembly of the flagellum in *P. putida* KT2440. The data reflect differences between process start (0 h) and 24 h incubation in the bio-electrochemical system. Non-significant differences are shown in red (Benjamini-Hochberg FDR > 0.05). *n* = 3. Table S5: Impact of anoxic-electrochemical conditions on the expression of genes and protein abundance, related to fatty acid metabolism in *P. putida* KT2440. The data reflect differences between process start (0 h) and 24 h incubation in the bio-electrochemical system. Non-significant differences are shown in red (Benjamini-Hochberg FDR > 0.05). *n* = 3. Figure S1: Summed fraction labelling (SFL) of amino acids derived from hydrolyzed *P. putida* KT2440 cells after 100 h incubation on [^13^C_6_] glucose in the bio-electrochemical system. Share of protein-bound (98.4%) and free intracellular amino acids (1.6%) (A). The calculation was based on a cellular protein content of 0.553 g g^− 1^ [92] and intracellular amino acid levels in *P. putida* [93]. The SFL data of selected proteinogenic amino acids are given below (B). Figure S2: Principal component analysis of the transcriptome data. 0 h (T0, circle), 24 h (BES T1, down-pointing triangle), 100 h (BES T2, up-pointing triangle). Figure S3: Venn diagram of proteome and transcriptome data at different time points. Significantly down- (blue) and upregulated (yellow) genes at 24 h (T1) and 100 h (T2) compared to the process start (T0) (A). Significantly lower (blue) and higher (yellow) abundant proteins at 24 h (T1), 100 h (T2), and 380 h (Tend) compared to the process start (T0). Figure S4: Volcano plot depicting the differences between process start (0 h) and 100 h incubation in bio-electrochemical system. Significantly down- (Log_2_(FC) < -2, p_adj_ < 0.05; blue) and upregulated (Log_2_(FC) > 2, p_adj_ < 0.05; yellow) genes. Non-significant differences are shown in grey (Benjamini-Hochberg FDR > 0.05). Figure S5: Regression analysis for the determination of acetate/glucose yield coefficients for the wild type P. putida *KT2440* (WT) as well as the mutants *ΔacsA-I ΔacsA II*, ∆*PP_5266*, Δ*aldB-I ΔaldB-II*, and Δs*cpC*. Figure S6: Additional data related to the BES processes of different acetate mutants shown in Fig. 8. The data shown comprise the profiles of lactate, succinate, and pyruvate over time (mM), as well as the cell concentration (OD_600_) over time. Figure S7: Additional data related to the BES process of *P. putida* ∆ *aldBI* ∆*aldBII*. The data comprise the current density j [mA/cm^2^], and the concentration of [Fe(CN)_6_]^3−^ over time.

Supplementary Material 3: Additional file 3: table S1-Change in protein abundance.

## Data Availability

Data is provided within the manuscript or supplementary information files. In addition, the transcriptomic data set is accessible via GEO (GSE266590). The proteome data set can be accessed at MassIVE (MSV000094887).
