# Supplementary material for: Systems biology of electrogenic Pseudomonas putida - multi-omics insights and metabolic engineering for enhanced 2-ketogluconate production
Source: Microb Cell Fact. 2024 Sep 11;23:246. doi: 10.1186/s12934-024-02509-8 (PMC11389600; doi:10.1186/s12934-024-02509-8)
Supplement: Supplementary file 1 — Supplementary Material 1: Additional file 1: Figure S1. BES cultivation of P.putida KT2440 (Fig. 1).The displayed data were corrected for the evaporation of water. Table S1: Mean values of measured data (uncorrected) and data corrected for the evaporation of water (corrected). (n = 4). [file 12934_2024_2509_MOESM1_ESM.pdf]

Additional file 1 to

Systems biology of electrogenic *Pseudomonas putida* - multi-omics insights and metabolic engineering for enhanced 2-ketogluconate production

Anna Weimer[1], Laura Pause[2], Fabian Ries[1], Michael Kohlstedt[1], Lorenz Adrian[3], Jens Krömer[2], Bin Lai[4], and Christoph Wittmann[1#]

[1] Institute of Systems Biotechnology, Saarland University, Saarbrücken, Germany

[2] Systems Biotechnology Group, Helmholtz Centre for Environmental Research - UFZ, Leipzig, Germany

[3] Department of Molecular Environmental Biotechnology, Helmholtz Centre for Environmental Research – UFZ, Leipzig, Germany

[4] BMBF Junior Research Group Biophotovoltaics, Helmholtz Centre for Environmental Research - UFZ, Leipzig, Germany

[#]Phone/FAX: +49 681 302 71970/71972, e-mail: christoph.wittmann@uni-saarland.de

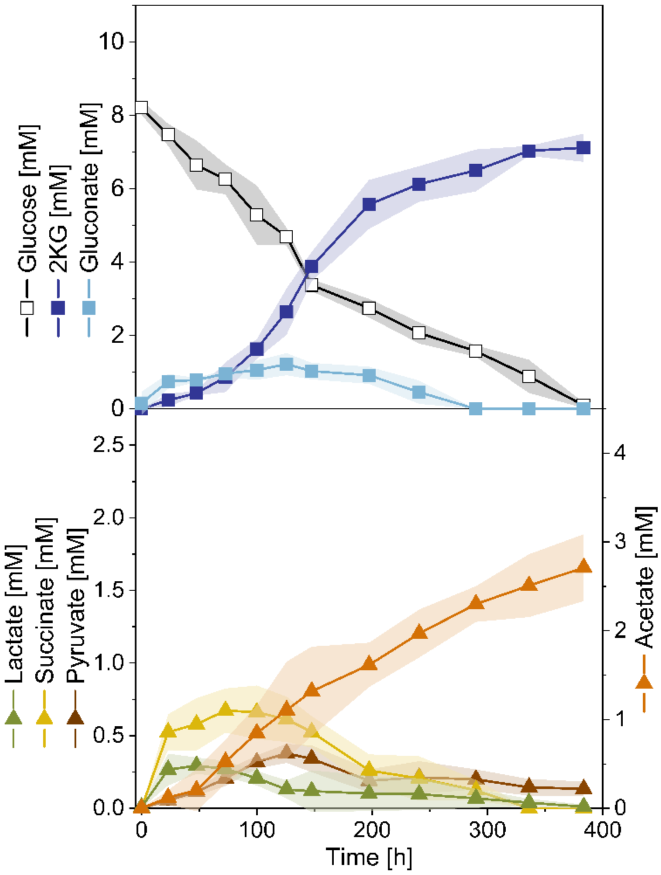

Figure S1. BES cultivation of *P. putida* KT2440 (Fig. 1). The displayed data were corrected for the evaporation of water.

Table S1: Mean values of measured data (uncorrected) and data corrected for the evaporation of water (corrected). (n=4).

Time	Glucose	2-Ketogluconate	Gluconate	Acetate	Pyruvate	Succinate	Lactate
h	mM	mM	mM	mM	mM	mM	mM
**Uncorrected**							
0	8.21	0.00	0.08	0.00	0.00	0.00	0.01
23.03	7.52	0.24	0.76	0.12	0.06	0.53	0.27
47.67	6.74	0.43	0.80	0.21	0.12	0.59	0.30
72.78	6.39	0.88	0.80	0.54	0.21	0.69	0.28
99.97	5.44	1.68	1.09	0.87	0.33	0.68	0.21
125.73	4.87	2.75	1.26	1.15	0.39	0.63	0.13
147.5	3.52	4.05	1.08	1.38	0.36	0.55	0.12
197.28	2.91	5.90	0.97	1.71	0.20	0.28	0.11
240.75	2.08	6.57	0.49	2.11	0.23	0.29	0.10
290.03	1.72	7.30	0.00	2.51	0.22	0.13	0.07
335.98	0.98	7.55	0.00	2.77	0.16	0.00	0.04
383.45	0.18	7.94	0.00	3.04	0.15	0.00	0.01
**Corrected**							
h	mM	mM	mM	mM	mM	mM	mM
0	8.21	0.00	0.08	0.00	0.00	0.00	0.01
23.03	7.47	0.24	0.75	0.12	0.05	0.52	0.27
47.67	6.65	0.43	0.79	0.2	0.11	0.58	0.29
72.78	6.26	0.86	0.96	0.53	0.21	0.67	0.27
99.97	5.28	1.63	1.06	0.85	0.32	0.66	0.21
125.73	4.70	2.65	1.22	1.11	0.38	0.61	0.13
147.5	3.37	3.88	1.03	1.32	0.34	0.53	0.12
197.28	2.75	5.57	0.92	1.62	0.19	0.26	0.10
240.75	2.07	6.13	0.46	1.97	0.21	0.21	0.10
290.03	1.58	6.5	0.00	2.3	0.2	0.12	0.07
335.98	0.89	7.03	0.00	2.51	0.14	0.00	0.04
383.45	0.08	7.12	0.00	2.71	0.13	0.00	0.01
