# Supplementary material for: Systems biology of electrogenic Pseudomonas putida - multi-omics insights and metabolic engineering for enhanced 2-ketogluconate production
Source: Microb Cell Fact. 2024 Sep 11;23:246. doi: 10.1186/s12934-024-02509-8 (PMC11389600; doi:10.1186/s12934-024-02509-8)
Supplement: Supplementary file 2 — Supplementary Material 2: Additional file 2: Table S1-List of primers used for genetic engineering. The overhangs for Gibson assembly are underscored. Table S2: Fatty acid composition of P. putida KT2440 at the start of the process (0 h) and after 100 h incubation in the bio-electrochemical system. The data are given in % of total fatty acids. Table S3: Impact of anoxic-electrochemical conditions on the expression of genes related to central carbon metabolism in P. putida KT2440. The data reflect significant differences between process start (0 h) and 24 h incubation in the bio-electrochemical system. n = 3. Table S4: Impact of anoxic-electrochemical conditions on the expression of genes related to assembly of the flagellum in P. putida KT2440. The data reflect differences between process start (0 h) and 24 h incubation in the bio-electrochemical system. Non-significant differences are shown in red (Benjamini-Hochberg FDR > 0.05). n = 3. Table S5: Impact of anoxic-electrochemical conditions on the expression of genes and protein abundance, related to fatty acid metabolism in P. putida KT2440. The data reflect differences between process start (0 h) and 24 h incubation in the bio-electrochemical system. Non-significant differences are shown in red (Benjamini-Hochberg FDR > 0.05). n = 3. Figure S1: Summed fraction labelling (SFL) of amino acids derived from hydrolyzed P. putida KT2440 cells after 100 h incubation on [13C6] glucose in the bio-electrochemical system. Share of protein-bound (98.4%) and free intracellular amino acids (1.6%) (A). The calculation was based on a cellular protein content of 0.553 g g− 1 [92] and intracellular amino acid levels in P. putida [93]. The SFL data of selected proteinogenic amino acids are given below (B). Figure S2: Principal component analysis of the transcriptome data. 0 h (T0, circle), 24 h (BES T1, down-pointing triangle), 100 h (BES T2, up-pointing triangle). Figure S3: Venn diagram of proteome and transcriptome data at differ [file 12934_2024_2509_MOESM2_ESM.pdf]

Additional file 2 to

Systems biology of electrogenic *Pseudomonas putida* - multi-omics insights and metabolic engineering for enhanced 2-ketogluconate production

Anna Weimer[1], Laura Pause[2], Fabian Ries[1], Michael Kohlstedt[1], Lorenz Adrian[3], Jens Krömer[2], Bin Lai[4], and Christoph Wittmann[1#]

[1] Institute of Systems Biotechnology, Saarland University, Saarbrücken, Germany

[2] Systems Biotechnology Group, Helmholtz Centre for Environmental Research - UFZ, Leipzig, Germany

[3] Department of Molecular Environmental Biotechnology, Helmholtz Centre for Environmental Research – UFZ, Leipzig, Germany

[4] BMBF Junior Research Group Biophotovoltaics, Helmholtz Centre for Environmental Research - UFZ, Leipzig, Germany

[#]Phone/FAX: +49 681 302 71970/71972, e-mail: christoph.wittmann@uni-saarland.de

Table S1. **List of primers used for genetic engineering**. The overhangs for Gibson assembly are underscored.

Name	Sequence (5'→3')	Application
**PP_5266_UP_fwd**	GAATTCGAGCTCGGTACCCGAAACCGAAGTAATTACCAAGAC	Amplification of upstream region for deletion of PP_5266
**PP_5266_UP_rev**	TGCGAATGGCCGCAACCCTTGGGGCCTCCTGAAACAT	Amplification of upstream region for deletion of PP_5266
**PP_5266_DW_fwd**	CGGATGTTTCAGGAGGCCCCAAGGGTTGCGGCCATTCG	Amplification of downstream region for deletion of PP_5266
**PP_5266_DW_rev**	GTCGACTCTAGAGGATCCCCATTGCCCCAGCGGTGATG	Amplification of downstream region for deletion of PP_5266
**acsA-I_UP_fwd**	GGTATTGCCGGGAAGGGTTACAGCCTTGCCGACGAAA	Amplification of upstream region for deletion of acsA-I
**acsA-I_UP_rev**	TGAATTCGAGCTCGGTACCCCCATCACGCGGTATTCGAGA	Amplification of upstream region for deletion of acsA-I
**acsA-I_DW_fwd**	GTCGACTCTAGAGGATCCCCGGTAACAGCTGCCCGATATG	Amplification of downstream region for deletion acsA-I
**acsA-I_DW_rev**	TTCGTCGGCAAGGCTGTAACCCTTCCCGGCAATACC	Amplification of downstream region for deletion acsA-I
**acsA-II_UP_fwd**	GCCAGATTTGCGGCCGCCGGGGCGGGTGGTCCTGCTCTT	Amplification of upstream region for deletion of acsA-II
**acsA-II_UP_rev**	TGAATTCGAGCTCGGTACCCTGCGCGCTAAAGGCCTCAAC	Amplification of upstream region for deletion of acsA-II
**acsA-II_DW_fwd**	GTCGACTCTAGAGGATCCCCCATTATCCGTAGGACGAGCCG	Amplification of downstream region for deletion acsA-II
**acsA-II_DW_rev**	CAAGAGCAGGACCACCCGCCCCGGCGGCCGCAAATCTG	Amplification of downstream region for deletion acsA-II
**aldB-I_UP_fwd**	GCGAAGAAGGCGACGCGGTGGTGTGTCTCCTTGGTATTGT	Amplification of upstream region for deletion of aldB-I
**aldB-I_UP_rev**	TGAATTCGAGCTCGGTACCCCTGGTGCAGGCTGTTCATTT	Amplification of upstream region for deletion of aldB-I
**aldB-I_DW_fwd**	GTCGACTCTAGAGGATCCCCCGACGACAGTTCGGCCAGCG	Amplification of downstream region for deletion aldB-I
**aldB-I_DW_rev**	ACAATACCAAGGAGACACACCACCGCGTCGCCTTCTTCG	Amplification of downstream region for deletion aldB-I
**aldB-II_UP_fwd**	GGTATTGCCGGGAAGGGTTACAGCCTTGCCGACGAAA	Amplification of upstream region for deletion of aldB-II
**aldB-II_UP_rev**	TGAATTCGAGCTCGGTACCCCCATCACGCGGTATTCGAGA	Amplification of upstream region for deletion of aldB-II
**aldB-II_DW_fwd**	GTCGACTCTAGAGGATCCCCGGTAACAGCTGCCCGATATG	Amplification of downstream region for deletion aldB-II
**aldB-II_DW_rev**	TTCGTCGGCAAGGCTGTAACCCTTCCCGGCAATACC	Amplification of downstream region for deletion aldB-II
**scpC_UP_fwd**	GTCGACTCTAGAGGATCCCCGGTCCTGGCCTTCATCATG	Amplification of upstream region for deletion of scpC
**scpC_UP_rev**	CGTTCCGTACCACATCCGGAGGATTGTTATCTCGGGCTACTG	Amplification of upstream region for deletion of scpC
**scpC_DW_fwd**	GTAGCCCGAGATAACAATCCTCCGGATGTGGTACGGAACG	Amplification of downstream region for deletion scpC
**scpC_DW_rev**	CTGAATTCGAGCTCGGTACCCGAGCTCACGTCGGATGTGG	Amplification of downstream region for deletion scpC

Table S2: **Fatty acid composition of *P. putida* KT2440 at the start of the process (0 h) and after 100 h incubation in the bio-electrochemical system.** The data are given in % of total fatty acids.

**Fatty acid**	**0 h**	**100 h**
10:0 3OH	2.0	1.6
12:0	4.4	6.2
12:0 2OH	1.0	1.4
12:1 3OH w7c	0.2	0.1
12:0 3OH	1.6	2.1
14:1 w7c	0.2	0.1
14:1 w5c	-	0.1
14:0	0.3	0.3
16:1 w7c	**30.6**	**7.0**
16:1 w7t	**2.3**	**14.8**
16:0	30.2	32.9
17:0 cyclo w7c	0.3	0.8
18:1 w7c	**25.7**	**12.6**
18:1 w7t	**-**	**17.8**
18:1 w5c	0.1	
18:0	1.1	2.1
Sum n:0	36.0	41.5
Sum n:0 OH	4.6	5.1
Sum n:1 cis	**56.8**	**19.9**
Sum n:0 cyclo	0.3	0.8
Sum n:1 trans	**2.3**	**32.6**
Degree of saturation	40.6	46.7
Average carbon chain length	16.1	16.2

Table S3: Impact of anoxic-electrochemical conditions on the expression of genes related to central carbon metabolism in *P. putida* KT2440. The data reflect significant differences between process start (0 h) and 24 h incubation in the bio-electrochemical system. n=3.

Metabolic pathway	Gene name	Locus tag	log2FC	adj. -value
**Glucose uptake**	*oprB-I*	PP_1019	-1.41	***1.75E-01***
	oprB-II	PP_1445	0.86	4.45E-02
	oprB-III	PP_3570	2.73	3.05E-05
	gtsA	PP_1015	-1.80	1.90E-02
	gtsB	PP_1016	-0.26	5.45E-02
	gtsC	PP_1017	-0.49	***3.37E-01***
	gtsD	PP_1018	-0.10	***8.95E-01***
	glk	PP_1011	0.15	***6.01E-01***
**Gluconate / 2-Ketogluconate formation / uptake**	*gcd*	PP_1444	-1.44	3.01E-03
	gnl	PP_1170	1.88	1.65E-04
	gadA / gdh	PP_3382	2.51	2.43E-04
	gadB / gdh	PP_3383	1.87	1.86E-04
	gadC / gdh	PP_3384	1.05	1.97E-04
	gad / gdh	PP_3623	-1.48	6.14E-03
	gad / gdh	PP_4232	2.94	8.33E-06
	gnuK	PP_3416	-0.48	***2.60E-01***
	gntT	PP_3417	0.19	***5.56E-01***
	kguT	PP_3377	0.39	***7.34E-02***
	kguK	PP_3378	1.39	***2.43E-01***
**Entner-Doudoroff pathway**	*edd*	PP_1010	3.58	3.61E-07
	eda	PP_1024	-0.80	***2.27E-01***
**Pentose Phosphate pathway**	*zwf-I*	PP_1022	-0.95	***5.71E-02***
	zwf-II	PP_4042	-0.77	4.60E-02
	zwf	PP_5351	0.68	3.15E-02
	pgl	PP_1023	-0.81	***1.46E-01***
	rpe	PP_0415	1.88	1.69E-03
	gnd	PP_4043	-0.39	***3.28E-01***
	tktA	PP_4965	1.25	2.35E-03
		PP_5367	1.10	***2.42E-01***
	tal	PP_2168	-1.30	5.20E-04
	rpiA	PP_5150	-0.08	***1.43E-01***
**Embden-Meyerhof-Parnas pathway**	*pgi-1*	PP_1808	-0.51	2.67E-02
	pgi-2	PP_4701	-0.23	***6.04E-01***
	fbp	PP_5040	-0.73	1.61E-02
	fda	PP_4960	-0.36	3.39E-02

		PP_2037	3.97	2.08E-03
		PP_2871	3.20	4.09E-06
		PP_3224	4.32	7.65E-06
	tpiA	PP_4715	-0.09	2.92E-02
	gap-I / gapA	PP_1009	1.62	1.81E-02
	gap-II / gapB	PP_2149	0.92	***3.13E-01***
		PP_0665	3.29	2.89E-06
		PP_3443	-0.90	2.52E-02
	pgk	PP_4963	0.88	***1.59E-01***
	pgm	PP_3578	0.46	***2.40E-01***
		PP_2243	3.19	2.44E-06
		PP_3923	1.58	3.56E-07
		PP_4450	0.01	3.89E-04
	pykA	PP_1362	-0.87	4.10E-02
	pykF	PP_4301	4.01	1.14E-06
	ppsA	PP_2082	-0.22	***3.14E-01***
		PP_2081	-0.01	7.48E-03
**Pyruvate dehydrogenase**	*acoA*	PP_0555	2.98	1.78E-06
	acoB	PP_0554	4.01	2.75E-06
	acoC	PP_0553	3.52	7.83E-05
	aceF	PP_0338	3.29	4.42E-06
	aceE	PP_0339	1.15	***2.55E-01***
**Citric acid cycle**	*gltA*	PP_4194	1.25	2.88E-02
	acnAI	PP_2112	-0.18	***1.89E-01***
	acnB	PP_2339	0.74	5.31E-03
	acnAII	PP_2336	0.25	1.10E-02
	icd	PP_4011	-2.55	1.09E-03
	idh	PP_4012	2.23	2.26E-02
	aceK	PP_4565	0.41	3.77E-03
	sdhA	PP_4191	-0.18	***7.92E-01***
	sdhB	PP_4190	-0.04	***5.87E-01***
	sdhD	PP_4192	-0.85	1.09E-02
	sdhC	PP_4193	-0.47	3.02E-02
	sucD	PP_4185	-0.44	***6.36E-01***
	sucC	PP_4186	-0.17	***8.58E-01***
	sucA	PP_4189	0.64	***5.43E-01***
	sucB	PP_4188	1.09	***3.25E-01***
	lpdG	PP_4187	1.07	***3.37E-01***
	fumC-I	PP_0944	3.43	1.86E-02
	fumC-II	PP_1755	-1.33	2.85E-02
		PP_0897	2.28	1.51E-03
		PP_2652	0.38	***9.11E-02***
	mdh	PP_0654	-0.99	4.69E-03
	mqo-I	PP_0751	1.58	7.89E-03

	mqo-II	PP_1251	2.84	4.64E-03
	mqo-III	PP_2925	-1.26	2.40E-03
		PP_3591	-0.59	2.79E-02
**Glyoxylate shunt**	*aceA*	PP_4116	3.15	6.84E-05
	glcB	PP_0356	1.71	7.03E-03
**Anaplerosis / Gluconeogenesis**	*ppc*	PP_1505	1.78	1.31E-03
	pycB	PP_5346	2.6	1.01E-03
	pycA	PP_5347	2.47	8.18E-05
	maeB	PP_5085	2.06	***5.63E-01***
**Acetate formation**	*acsA-I*	PP_4487	2.16	1.69E-02
	acsA-II	PP_4702	0.91	***2.03E-01***
	aldB-I	PP_0545	1.27	***1.55E-01***
	aldB-II	PP_2680	2.81	1.08E-02
	scpC	PP_0154	2.93	2.04E-05
		PP_5266	2.26	7.53E-05
**Lactate formation**	*lldD*	PP_4736	2.22	1.37E-03
**C4-dicarboxylate transport**	*dctA-I*	PP_1188	2.21	7.39E-04
	dctA-II	PP_2056	2.00	***6.76E-02***
	dctA-III	PP_2255	-0.85	***1.82E-01***
**Acetate symport**	*actP-I*	PP_1743	2.64	2.78E-03
	actP-II	PP_2797	1.42	1.09E-02
	actP-III	PP_3272	4.06	5.27E-07

Table S4: Impact of anoxic-electrochemical conditions on the expression of genes related to assembly of the flagellum in *P. putida* KT2440. The data reflect differences between process start (0 h) and 24 h incubation in the bio-electrochemical system. Non-signficant differences are shown in red (Benjamini-Hochberg FDR >0.05). n=3.

Metabolic pathway	Gene name	Locus tag	log2FC	Adj. p-value
Flagellar assembly	*fliE*	PP_4370	-0.99	1.00E-02
	fliF	PP_4369	-0.01	2.55E-02
	fliG	PP_4368	-1.07	1.97E-03
	fliH	PP_4367	-0.09	0.20
	fliI	PP_4366	-0.76	0.22
	fliJ	PP_4365	0.26	0.10
	fliK	PP_4361	-0.85	0.13
	fliL	PP_4359	-0.29	8.95E-05
		PP_5209	-1.95	7.54E-06
	fliM	PP_4358	-0.14	4.17E-03
	fliN	PP_4357	-0.01	1.74E-02
	fliO	PP_4356	0.98	3.97E-03
	fliP	PP_4355	0.76	0.19
	fliQ	PP_4354	0.20	1.42E-02
	flhB	PP_4352	1.71	6.77E-05
	flgA	PP_4394	-0.80	1.49E-02
	flgB	PP_4391	-2.41	5.41E-05
	flgC	PP_4390	-3.14	5.65E-05
	flgD	PP_4389	-2.78	4.26E-06
	flgE	PP_4388	-2.77	2.30E-05
	flgF	PP_4386	-1.75	7.05E-05
	flgG	PP_4385	-1.62	1.29E-04
	flgH	PP_4384	-2.02	3.21E-05
	flgI	PP_4383	-1.03	6.85E-03
	flgJ	PP_4382	-1.28	2.26E-03
	flgK	PP_4381	-1.10	0.17
	flgL	PP_4380	-2.04	2.21E-03
		PP_1087	-1.39	4.80E-03
	fliC	PP_4378	-1.60	9.61E-03
	fliD	PP_4376	-3.01	2.90E-06
	fliS	PP_4375	-2.74	3.68E-05
	fliT	PP_4374	-2.64	5.31E-06

	motA	PP_4905	-0.56	8.55E-02
		PP_4335	-0.41	0.17
	flgM	PP_4395	-1.40	2.41E-02
		PP_4396	-1.40	0.10
	fliY	PP_0227	-1.08	2.35E-02
		PP_5157	0.94	3.80E-03
	fleQ	PP_4373	-0.66	6.62E-03
	rpoN	PP_0952	-0.49	0.17
	atoC	PP_4371	-0.09	0.15
	fliA	PP_4341	-0.89	4.40E-02

Table S5: Impact of anoxic-electrochemical conditions on the expression of genes and protein abundance, related to fatty acid metabolism in *P. putida* KT2440. The data reflect differences between process start (0 h) and 24 h incubation in the bio-electrochemical system. Non-signficant differences are shown in red (Benjamini-Hochberg FDR >0.05). n=3.

Metabolic pathway	Gene name	Locus Tag	Log2FC - T1 Transcriptome	Log2FC - T1 Proteome
**Fatty acid de novo synthesis**	*accA*	PP_1607	-1.59	0.42
	accB	PP_0559	-1.84	
	accC	PP_0558	-0.73	0.74
	atoB	PP_3123	-2.32	
	fabB	PP_4175	-0.38	
	fabF	PP_1916	1.69	0.8
	fabD	PP_1913	0.1	-2.05
	fabG	PP_1914	-0.94	
	fabZ	PP_1602	0.16	1.1
	fabA	PP_4174	-1.06	
	aacS	PP_3071	0.52	
	acpP	PP_1915	-0.45	-2.59
	fabH	PP_4379	-0.46	
**ß-oxidation**	*fadA*	PP_2051	2.30	
	fadB	PP_2136	1.31	
	fadBA	PP_2214	-0.81	
	fadE	PP_1893	-0.53	
	fadD-I	PP_4549	0.65	
	fadD-II	PP_4550	-0.40	
	yqeF	PP_4636	-1.15	
	acd	PP_2216	-1.89	
	paaF	PP_3284	4.38	
	paaH	PP_3282	4.43	-0.37
	pcaF-I	PP_1377	4.46	
	pcaF-II	PP_2137	0.91	
	bktB	PP_3754	-0.23	
**Methylcitrate cycle**	*mmgF*	PP_2334	-2.04	
	prpC	PP_2335	-1.47	
	acnA-II	PP_2336	0.25	
	prpF	PP_2337	0.44	
	prpD	PP_2338	0.37	
	cti	PP_2376	2.15	

A

B

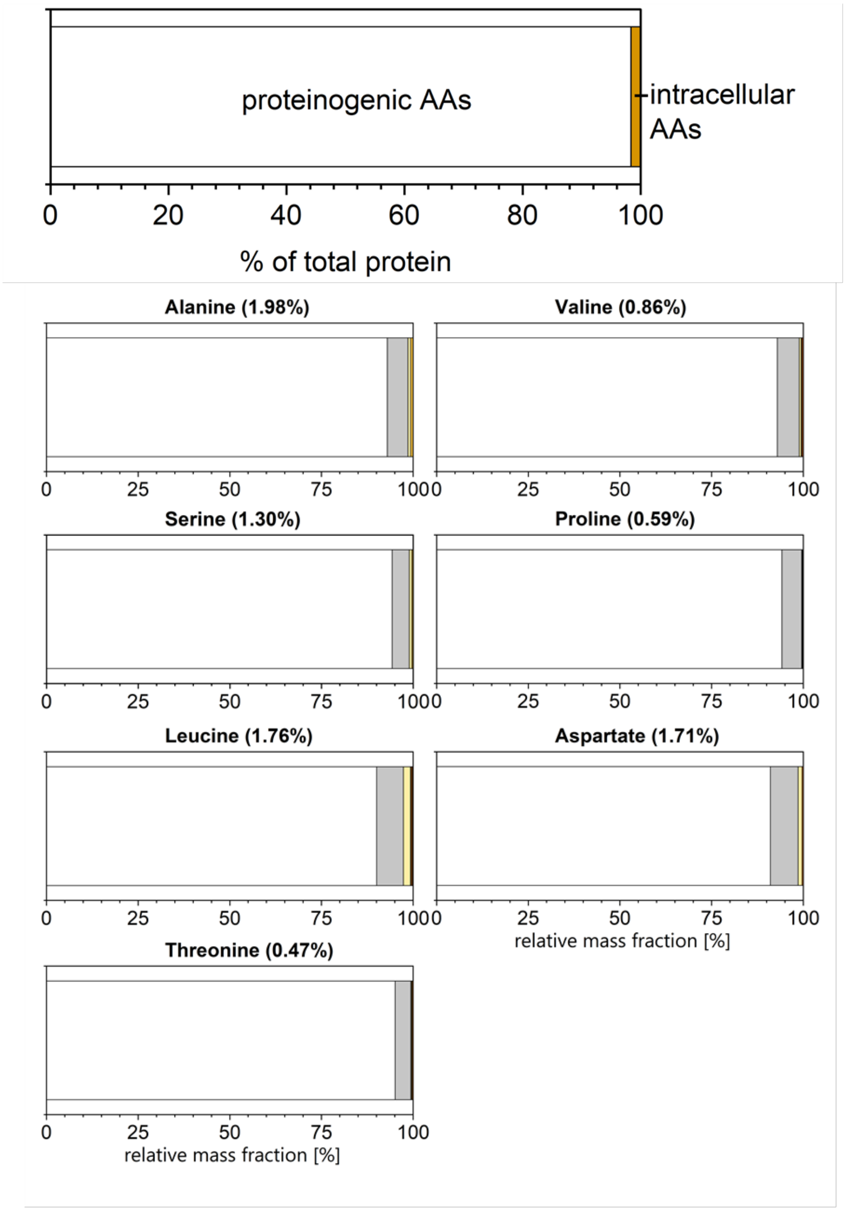

Figure S1: Summed fraction labelling (SFL) of amino acids derived from hydrolyzed *P. putida* KT2440 cells after 100 h incubation on [$^{13}C_6$] glucose in the bio-electrochemical system. Share of protein-bound (98.4%) and free intracellular amino acids (1.6%) (A). The calculation was based on a cellular protein content of 0.553 g g^{-1} [1] and intracellular amino acid levels in *P. putida* [2]. The SFL data of selected proteinogenic amino acids are given below (B).

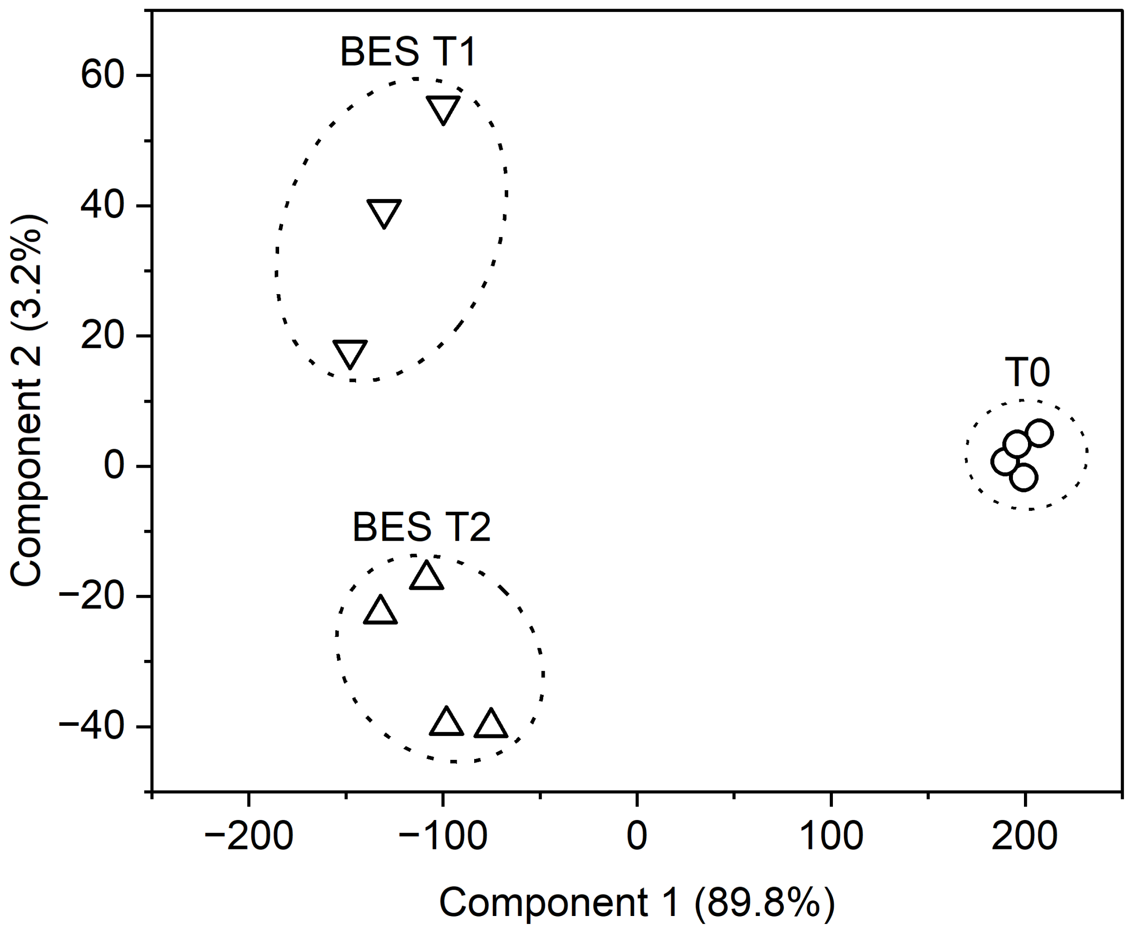

Figure S2: Principal component analysis of the obtained transcriptome data. T0 (preculture; circle), T1 (BES 24 h; down-pointing triangle), T2 (BES 100 h; up-pointing triangle).

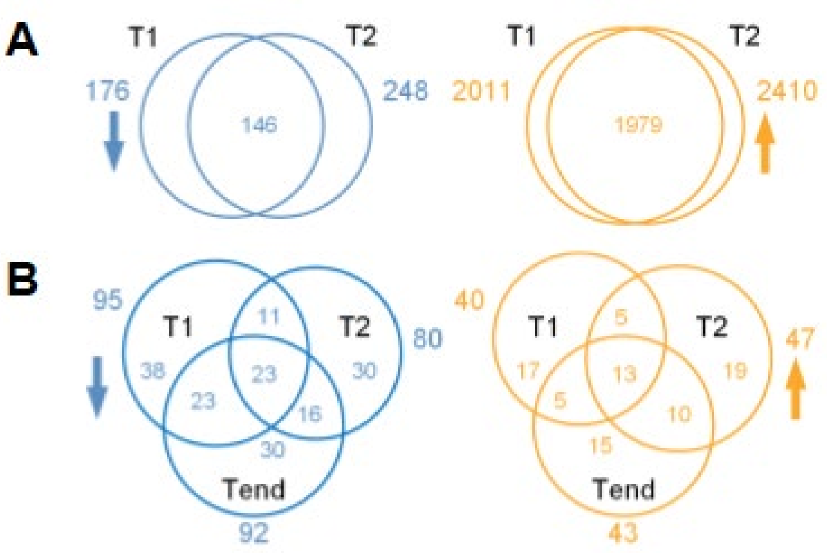

Figure S3: Venn diagram of proteome and transcriptome data at different time points. Significantly down- (blue) and upregulated (yellow) genes at T1 (24 h) and T2 (100h) compared to T0 (pre-culture) (A). Signifcantly lower (blue) and higher (yellow) abundant proteins at T1 (24 h), T2 (100h) and Tend (380h) compared to T0 (pre-culture).

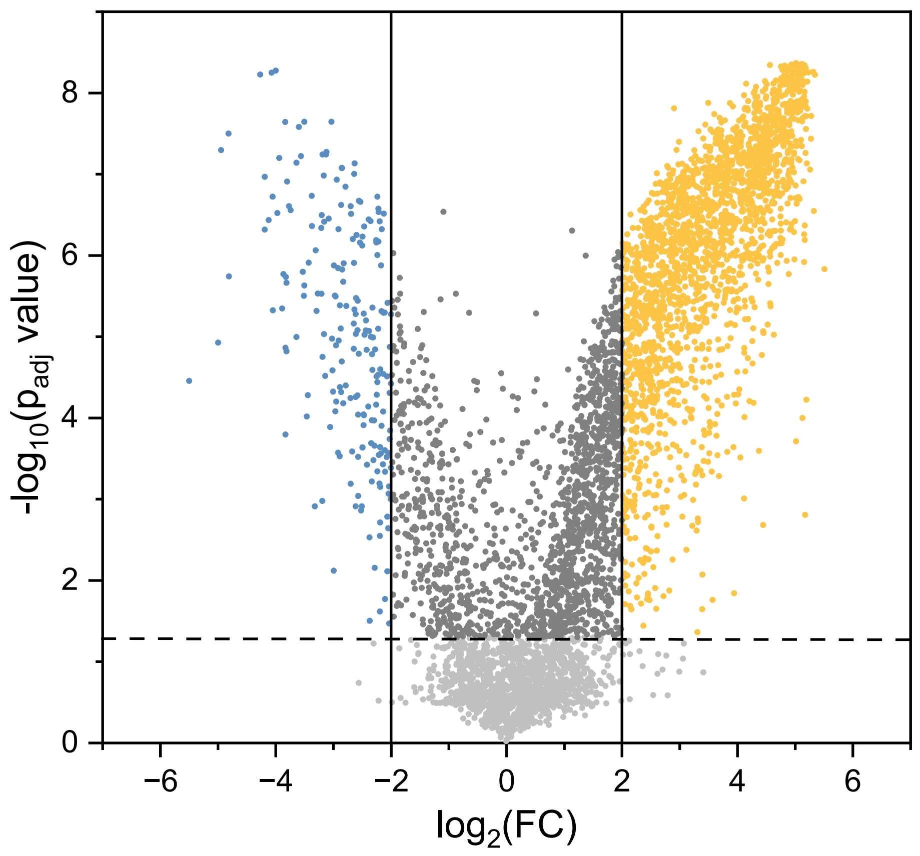

Figure S4: Volcano plot T2 vs T0. Significantly down- (Log2(FC)<-2, p_{adj}<0.05; blue) and upregulated (Log2(FC)>2, p_{adj}<0.05; yellow) genes at T2 (100 h) compared to T0 (0 h).

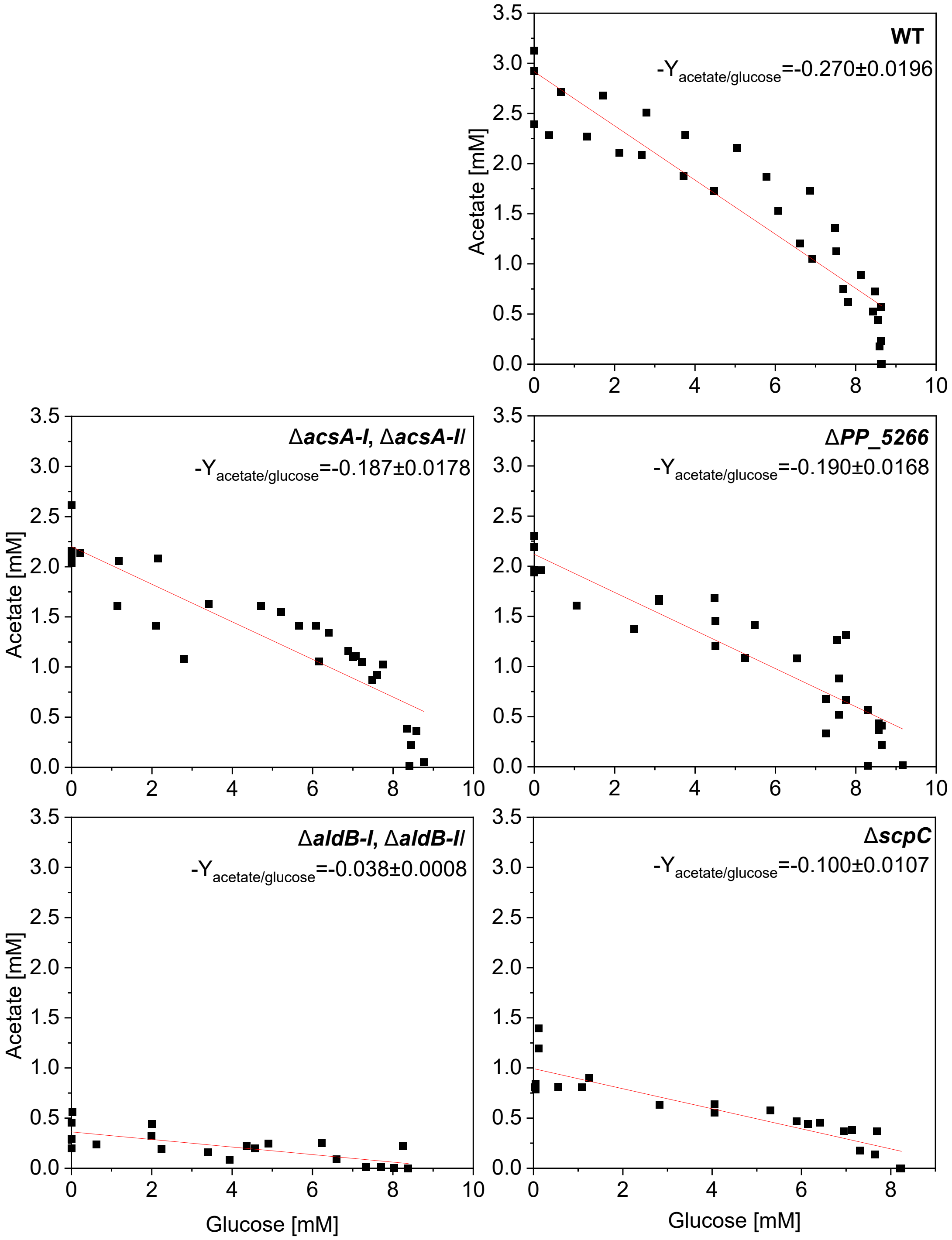

Figure S5: Regression analysis for the determination of acetate/glucose yield coefficients for the wild type P. putida *KT2440* (WT) as well as the mutants Δ*acsA-I* Δ*acsA II*, Δ*PP5266*, Δ*aldB-I* Δ*aldB-II*, and Δ*scpC* in BES.

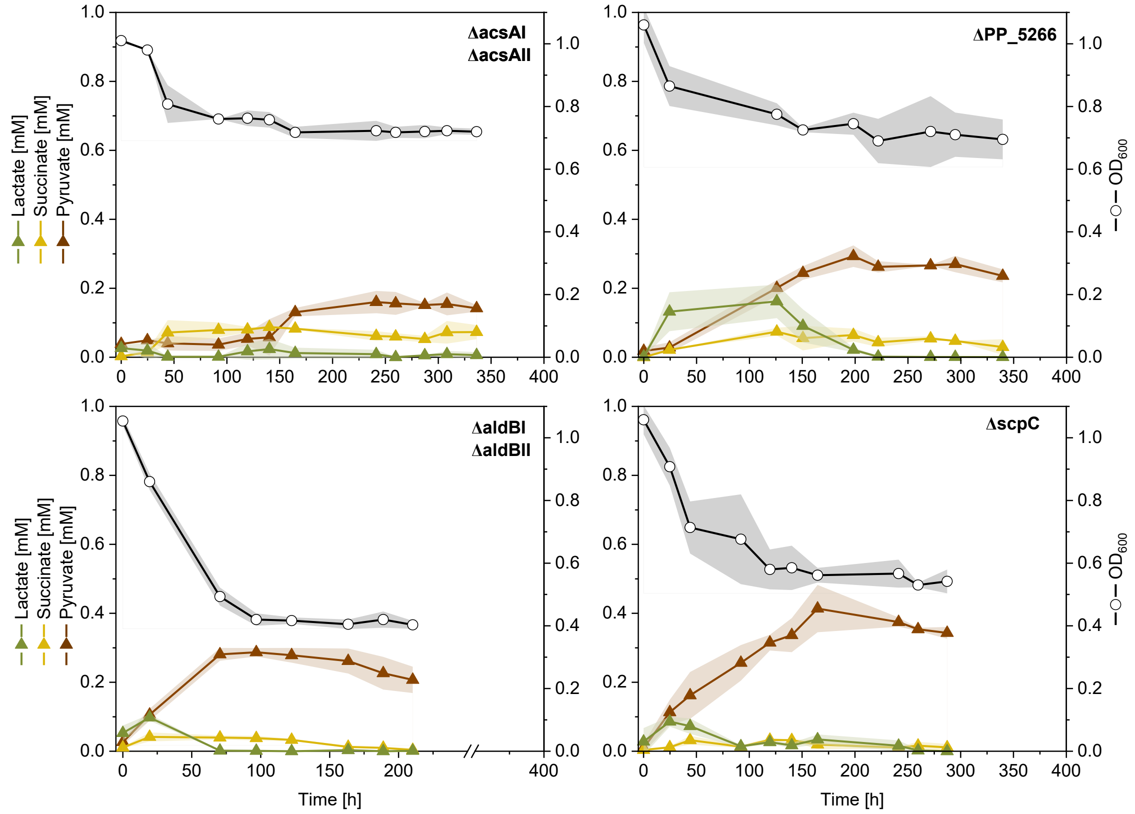

Figure S6: Additional data related to the BES processes of different acetate mutants shown in Fig. 8. The data comprise the profiles of lactate, succinate, and pyruvate over time (mM), as well as the cell concentration (OD_{600}) over time.

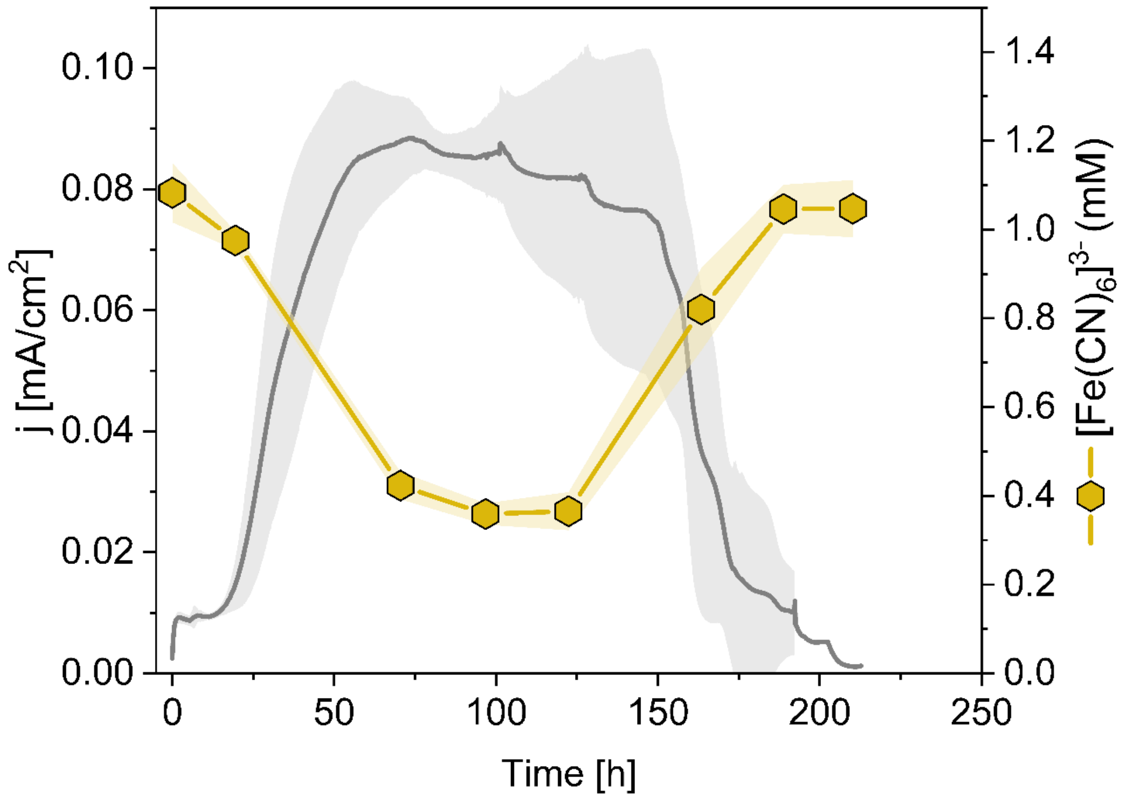

Figure S7: Additional data related to the BES process of *P. putida* ∆*aldBI* ∆*aldBII*. The data comprise the current density j [mA/cm^2], and the concentration of $[Fe(CN)_6]^{3-}$ over time.

Literature

1. van Duuren JB, Puchalka J, Mars AE, Bucker R, Eggink G, Wittmann C, Dos Santos VA: **Reconciling *in vivo* and *in silico* key biological parameters of *Pseudomonas putida* KT2440 during growth on glucose under carbon-limited condition.** *BMC Biotechnol* 2013, **13:**93.
2. Bolten CJ, Kiefer P, Letisse F, Portais JC, Wittmann C: **Sampling for metabolome analysis of microorganisms.** *Anal Chem* 2007, **79:**3843-3849.
